# Phagocytic Receptor CED-1 Initiates a Signaling Pathway for Degrading Engulfed Apoptotic Cells

**DOI:** 10.1371/journal.pbio.0060061

**Published:** 2008-03-18

**Authors:** Xiaomeng Yu, Nan Lu, Zheng Zhou

**Affiliations:** 1 Verna and Marrs McLean Department of Biochemistry and Molecular Biology, Baylor College of Medicine, Houston, Texas, United States of America; 2 Program in Developmental Biology, Baylor College of Medicine, Houston, Texas, United States of America; Max-Planck-Institute of Cell Biology and Genetics, Germany

## Abstract

Apoptotic cells in animals are engulfed by phagocytic cells and subsequently degraded inside phagosomes. To study the mechanisms controlling the degradation of apoptotic cells, we developed time-lapse imaging protocols in developing *Caenorhabditis elegans* embryos and established the temporal order of multiple events during engulfment and phagosome maturation. These include sequential enrichment on phagocytic membranes of phagocytic receptor cell death abnormal 1 (CED-1), large GTPase dynamin (DYN-1), phosphatidylinositol 3-phosphate (PI(3)P), and the small GTPase RAB-7, as well as the incorporation of endosomes and lysosomes to phagosomes. Two parallel genetic pathways are known to control the engulfment of apoptotic cells in C. elegans. We found that null mutations in each pathway not only delay or block engulfment, but also delay the degradation of engulfed apoptotic cells. One of the pathways, composed of CED-1, the adaptor protein CED-6, and DYN-1, controls the rate of enrichment of PI(3)P and RAB-7 on phagosomal surfaces and the formation of phagolysosomes. We further identified an essential role of RAB-7 in promoting the recruitment and fusion of lysosomes to phagosomes. We propose that RAB-7 functions as a downstream effector of the CED-1 pathway to mediate phagolysosome formation. Our work suggests that phagocytic receptors, which were thought to act specifically in initiating engulfment, also control phagosome maturation through the sequential activation of multiple effectors such as dynamin, PI(3)P, and Rab GTPases.

## Author Summary

Cells undergoing programmed cell death, or apoptosis, within an animal are swiftly engulfed by phagocytes and degraded inside phagosomes, vesicles in which the apoptotic cell is bounded by the engulfing cell's membrane. Little is known about how the degradation process is triggered and controlled. We studied the degradation of apoptotic cells during the development of the nematode Caenorhabditis elegans. Aided by a newly developed live-cell imaging technique, we identified multiple cellular events occurring on phagosomal surfaces and tracked the initiation signal to CED-1, a phagocytic receptor known to recognize apoptotic cells and to initiate their engulfment. CED-1 activates DYN-1, a large GTPase, which further activates downstream events, leading intracellular organelles such as endosomes and lysosomes to deliver to phagosomes various molecules essential for the degradation of apoptotic cells. As well as establishing a temporal order of events that lead to the degradation of apoptotic cells, the results suggest that phagocytic receptors, in addition to initiating phagocytosis, promote phagosome maturation through the sequential activation of multiple effector molecules.

## Abstract 3

The authors have identified multiple cellular events leading to the degradation of engulfed apoptotic cells in the nematode*C. elegans*, and found that CED-1, a phagocytic receptor thought to specifically control apoptotic-cell engulfment, activates a signaling pathway that initiates phagosome maturation.

## Introduction

**Figure 1:**
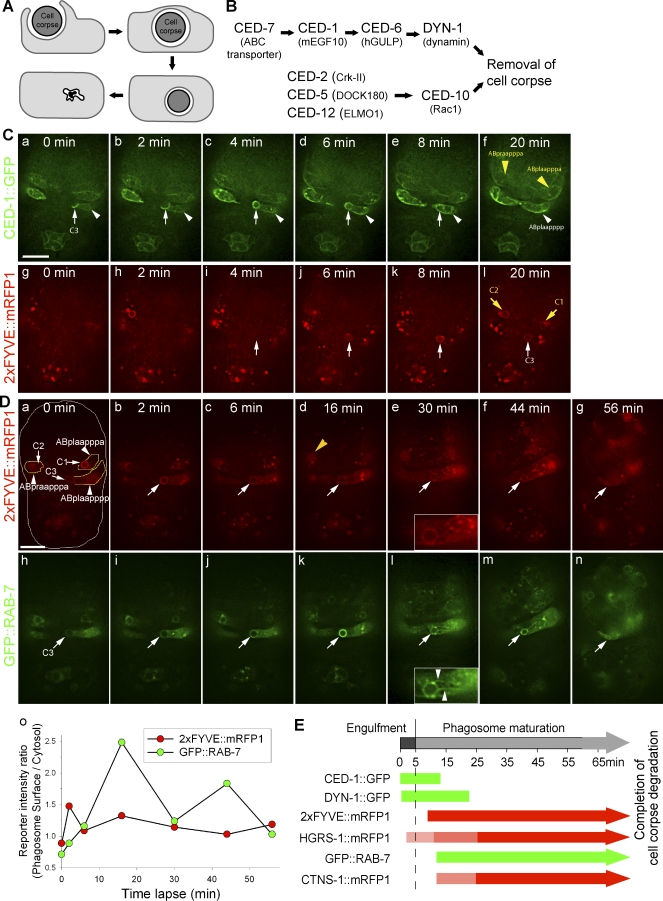
PI(3)P and RAB-7 Are Sequentially Enriched on the Surface of Maturing Phagosomes Containing Apoptotic Cells (A) Diagram of phagocytosis and the subsequent degradation of a cell corpse. (B) The two partially redundant and parallel pathways that control cell-corpse removal in C. elegans. Names in parentheses indicate mammalian homologs. (C and D) Time-lapse images of wild-type embryos coexpressing P*_ced-1_ced-1::gfp* / P*_ced-1_2xFYVE::mrfp1* (C) and P*_ced-1_2xFYVE::mrfp1* / P*_ced-1_gfp::rab-7* (D). Recording started at approximately 320 min after first cleavage. Anterior is to the top. Ventral faces readers. Scale bars indicate 10 μm. (C) Cell corpse C3 (white arrow) is engulfed by ABplaapppp (white arrowhead). Yellow arrows and arrowheads indicate C1 and C2 and their corresponding engulfing cells. “0 min” represents when CED-1::GFP is first detected on the budding pseudopods. (D) Time-lapse images of signals around C3 (arrows) during its degradation. “0 min” represents when ABplaapppp fully internalized C3. Arrows indicate C1, C2, and C3 in (a), of which the corresponding engulfing cells are outlined and indicated by arrowheads. Insets in (e) and (l) show an amplified view of the region surrounding C3; arrowheads indicate tubular structures. The yellow arrowhead in (d) depicts the nucleus of ABpraapppa, which has engulfed C2. (o) The relative reporter signal intensity measured from (a) to (n) is plotted over time. (E) Summary of the temporal order of each reporter detected on the surfaces of phagocytic cups and phagosomes. Data represent means obtained from time-lapse recording of multiple C1, C2, and C3 cell corpses (see text for actual numbers). “0 min” represents when budding pseudopods are first detected by CED-1::GFP. 2xFYVE, HGRS-1, RAB-7, and CTNS-1 reporters localize to phagosome surface until the cell corpse is fully degraded. The conversion of pale to deep pink colors of HGRS-1::mRFP1 and CTNS-1::mRFP1 indicates that starting from an initial weak signal, the signal intensity gradually increases.

An engulfing cell recognizes an apoptotic cell through phagocytic receptor(s) and extends thin pseudopods around it to generate a phagocytic cup. The fusion of growing pseudopods and scission of a vacuole containing the apoptotic cell from the plasmalemma generate a phagosome inside the host cell (Figure 1A) [1]. The swift engulfment (phagocytosis) and subsequent degradation of apoptotic cells inside phagosomes eliminate dying cells before they release any potentially harmful contents, and actively prevent tissue injury, inflammatory responses, and autoimmune diseases [2]. Inefficient degradation of the components of engulfed apoptotic cells, such as nuclear DNA, similar to inefficient engulfment, results in severe inflammatory and autoimmune responses [3,4].

Despite its importance, the molecular mechanisms driving the degradation of apoptotic cells remain largely unknown. Our current knowledge of phagosome maturation, the process that results in the complete degradation of phagosomal contents, is primarily obtained through the studies of phagosomes containing opsonized foreign pathogens or undigestible latex beads. Early during phagosomal maturation, signaling molecules such as Rab5 and PI(3)P are observed to be enriched on nascent phagosomes and act to recruit multiple downstream factors [5]. Early and late endosomes and lysosomes continue to fuse with phagosomes, and provide necessary lipids and proteins for phagosome maturation. Phagosome lumen is gradually acidified. The final state, known as a phagolysosome, is characterized by its abundance of acid hydrolases and an acidic pH environment [5,6]. The maturation of phagosomes containing apoptotic cells, by contrast, is less characterized, and may display distinct mechanistic features. Macrophages that internalize foreign pathogens secrete proinflammatory cytokines that induce inflammatory responses, whereas those engulfing apoptotic cells secrete anti-inflammatory cytokines, indicating that the same type of phagocytes elicit different responses to different phagocytic targets [2,7].

Rab GTPases are important regulators of many vesicle trafficking events. Rab7 has been implicated in the endocytic pathway, lysosomes biogenesis, and phagosomal maturation [5]. In both mammalian and *Dictyostelium* cells that ingest latex beads, the overexpression of a dominant-negative form of Rab7 (Rab7(DN)) blocks the phagosomal acquisition of lysosomal contents [8,9]. It remains to be elucidated, however, whether the defect in phagolysosome formation is a secondary consequence of the defects in lysosomal biogenesis and/or functions. In addition, although the enrichment of Rab7 was observed on phagosomes containing apoptotic cells [10], whether Rab7 is required for the processing of engulfed apoptotic cells and how Rab7 is activated by upstream signals remain unknown.

During the development of Caenorhabditis elegans hermaphrodites, 131 somatic cells and 300–500 germ cells undergo programmed cell death [11]. These apoptotic cells (referred to as cell corpses) are distinguishable from living cells as highly refractile discs under a Nomarski differential interference contrast (DIC) microscope [12]. In C. elegans, cell corpses are removed by their neighboring cells [11]. Genetic screens have identified two parallel and partially redundant pathways that regulate the engulfment of cell corpses (Figure 1B) [1,11,13,14]. In one pathway, the phagocytic receptor cell death abnormal 1 (CED-1) is an engulfing cell-specific, single-pass transmembrane protein that recognizes neighboring cell corpses, clusters to the growing phagocytic cups, and initiates pseudopod extension [15]. The large GTPase DYN-1 (dynamin) acts downstream of CED-1 and CED-6, a candidate adaptor for CED-1, to control the delivery of intracellular vesicles to both phagocytic cups and phagosomes [1]. In the other pathway, CED-5/Dock180, CED-2/CrkII, and CED-12/ELMO1 act together to regulate CED-10/Rac1 GTPase and promote cell surface extension during engulfment [11]. CED-10 may also mediate certain activities for the CED-1 pathway [16].

Previously, transmission electron microscopy (TEM) analyses indicated that those cell corpses that persisted for hours in *ced-1*, *-6*, *-7*, *-2*, *-5*, *-10*, and *-12* mutants were mostly unengulfed. It was thus proposed that these *ced* genes specifically control the engulfment of cell corpses [15,17,18]. However, none of the corresponding *ced* mutants completely block engulfment, since even in the strongest single engulfment mutants, such as *ced-1* or *ced-5* mutants, only approximately 25% of embryonic apoptotic cells persist for longer than 5 h [1]. Previous TEM analyses, which only characterized the terminal mutant phenotypes hours after the apoptosis events, were unable to determine whether the rest of cell corpses are engulfed, or to detect defects in the kinetics of engulfment and/or phagosome maturation.

Recently, we developed a time-lapse imaging protocol and used it to characterize the function of DYN-1 in developing embryos [1]. We found that in engulfing cells, DYN-1 was recruited to the surface of phagocytic cups and phagosomes in a CED-1–, CED-6–, and CED-7–dependent manner. DYN-1 promoted the recruitment and fusion of endosomes to phagocytic membranes, events that provide lipid and protein material to support pseudopod extension and phagosome maturation [1]. These results suggest that the engulfment and degradation of apoptotic cells may share common regulatory mechanisms. Like *dyn-1* mutants, *ced-1* null mutant embryos display defects in the incorporation of early endosomes to phagocytic cups and phagosomes [1], suggesting that CED-1, and perhaps other pathway components, may also control phagosome maturation in addition to engulfment.

To test the above hypothesis and to further identify the mechanism controlling the degradation of cell corpses, we developed multiple markers and further expanded the scope of our time-lapse imaging assays to monitor the processes of engulfment and phagosome maturation as well as the cellular events occurring during these processes in living embryos. We identified novel functions of both engulfment pathways in regulating the degradation of engulfed cell corpses. We further elucidate that the signaling pathway led by CED-1 promotes phagolysosome formation through activating RAB-7, whose function is essential for promoting the recruitment and fusion of lysosomes to phagosomes.

## Results

### PI(3)P and RAB-7 Are Sequentially Enriched on Phagosomal Surfaces

We examined the maturation process of phagosomes containing apoptotic cells in C. elegans. Previously, we reported the transient recruitment of DYN-1 and the incorporation of early endosomes, which are labeled with HGRS-1, the worm homolog of mammalian endosomal protein Hrs, to the surface of phagosomes [1]. Here, we examined two additional molecular events on phagosomal surfaces: the accumulation of PI(3)P [19,20] and the recruitment of small GTPase Rab7 [8]. We monitored both events in developing embryos using modified versions of an established protocol (Materials and Methods) [1]. In all time-lapse experiments described below, we chose to follow the engulfment and degradation of three particular apoptotic somatic cells, C1, C2, and C3, which are located near each other on the ventral side of an embryo and die almost simultaneously during embryogenesis, at approximately 330 min past the first cell division (the first cleavage) (Figure 1C(l) and 1D(a)). ABplaapppa, ABpraapppa, and ABplaapppp, three ventral hypodermal cells, engulf C1, C2, and C3, respectively (Figure 1C(f) and 1D(a)), during their extension to the ventral midline [1]. In addition, all green fluorescent protein (GFP) or monomeric red fluorescence protein (mRFP1) [21] tagged reporters were expressed specifically in engulfing cells under the control of P*_ced-1_*, the *ced-1* promoter [15].

The FYVE domain of C. elegans EEA-1, in a tandem repeat, specifically associates with PI(3)P [22]. The FYVE-FYVE::mRFP1 reporter was detected primarily in cytoplasm as bright puncta, consistent with its endosomal localization (Figure 1C) [1]. The 2xFYVE markers were also evenly distributed in nuclei (Figure 1D(d)). In engulfing cells, we observed bright mRFP1 circles surrounding cell corpses (Figure 1C and 1D). Consistent results have been obtained independently [23]. In embryos that coexpressed P*_ced-1_ ced-1::gfp* and P*_ced-1_ 2xFYVE::mrfp1*, CED-1::GFP, but not 2xFYVE::mRFP1, was enriched on extending pseudopods (Figure 1C). Strikingly, bright mRFP1 circles appeared on phagosomal surfaces within 4.1 ± 1.0 min (*n* = 16) after the closure of pseudopods (Figure 1C(d), 1C(j), and 1E), indicating that PI(3)P is specifically presented on the surface of nascent phagosomes. Whereas the CED-1::GFP signal rapidly disappeared from phagosomal surface after the enclosure of the phagocytic cup (within 8.4 ± 2.0 min, *n* = 17) (Figure 1C and 1E), PI(3)P remained on a phagosome until its content was completely degraded (Figure 1D and 1E). PI(3)P thus labels the surface of phagosomes for almost their entire duration.

*C. elegans rab-7* encodes RAB-7, a likely ortholog of human Rab7 (Figure S1A and Text S1). In wild-type animals expressing P*_ced-1_ gfp::rab-7*, a functional GFP::RAB-7 was detected in the cytoplasm, and a portion of it is enriched on cytoplasmic puncta (Figures S1B and S3D). This punctate localization pattern is consistent with previous reports indicating that RAB-7 is localized to late endosomes and lysosomes [24,25]. In both embryos and adult hermaphrodite gonads, we observed robust GFP::RAB-7 signals around cell corpses (Figure S1C). Quantitative measurements of time-lapse images (Materials and Methods) indicated that the intensity of the GFP signal on phagosomal surfaces could reach as high as 2.5 times that in the cytosol of the same engulfing cell (Figure 1D(o)). In a time-lapse recording of embryos that coexpress GFP::RAB-7 and 2xFYVE::mRFP1, RAB-7 was recruited to the surface of a phagosome approximately 3 min after PI(3)P appeared on the same phagosome (Figure 1D and 1E). After the completion of engulfment, yet prior to the recruitment of RAB-7, a nascent phagosome appeared as a dark hole surrounded by evenly distributed GFP::RAB-7 in the engulfing cell cytoplasm (Figure 1D(h) and 1D(i)). Like 2xFYVE::mRFP1, GFP::RAB-7 persisted on the surface of a phagosome until the phagosome disappeared (Figure 1D and 1E, and Video S1).

**Figure 2:**
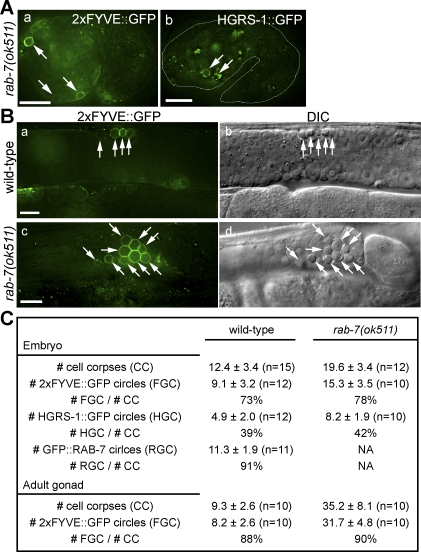
RAB-7 Is Not Required for the Enrichment of PI(3)P on Phagosomal Surfaces or the Incorporation of HGRS-1–Labeled Early Endosomes to Phagosomes Scale bars indicate 10 μm. Anterior is to the left. Ventral is to the bottom. Arrows indicate phagosomes containing cell corpses. (A) 2xFYVE::GFP (a) and HGRS-1::GFP (b) signals in 1.5-fold stage *rab-7(ok511)* (m^−^z^−^) embryos. White lines outline the shape of the embryos (b). (B) 2xFYVE::GFP signal in wild-type and *rab-7(ok511)* (m^+^z^−^) gonads of adult hermaphrodites staged at 48 h post-L4. (C) The numbers of cell corpses detected by DIC, 2xFYVE::GFP, and HGRS-1::GFP in gonads of adult hermaphrodites staged 48 h post-L4 and in 1.5-fold embryos. NA, not applicable due to the *gfp::rab-7* transgene rescuing the *rab-7(ok511)* (m^−^z^−^) phenotype. Data are presented as mean ± SD. *n*, number of animals scored.

To determine whether the enrichment of RAB-7 and PI(3)P was restricted to phagosomes containing C1, C2, and C3, we examined wild-type 1.5-fold to 2-fold stage embryos, which were at 420–460 min after first cleavage [1]. We found that 91% and 73% of cell corpses distinguished under DIC optics are labeled with GFP::RAB-7 and 2xFYVE::GFP, respectively (Figures 2C). These results indicate that both PI(3)P and RAB-7 are enriched on the surface of all phagosomes, and suggest that they may play important roles during phagosome maturation.

### Loss of *rab-7* Function Results in Maternal-Effect Embryonic Lethality and the Accumulation of a Large Number of Cell Corpses in the Body

**Figure 3:**
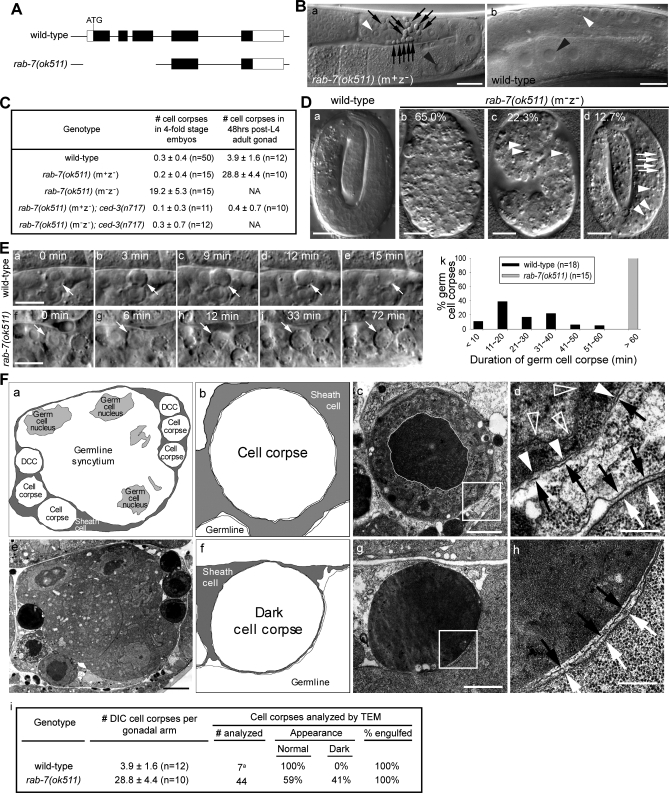
*rab-7(ok511)* Mutant Animals Accumulate Engulfed Cell Corpses and Are Maternal-Effect Embryonic Lethal (A) *rab-7* gene structure and the deletion in *rab-7(ok511*) allele. The coding and noncoding regions of exons are shown as black and white boxes, respectively. The single lines between exons are introns. (B) DIC images of *rab-7(ok511)* (m^+^z^−^) (a) and wild-type (b) gonads in adult hermaphrodites at 48 h post-L4 stage. Anterior is to the left. Ventral is to the bottom. Arrows indicate cell corpses. White and black arrowheads indicate germ cell and oocyte nuclei, respectively. Scale bars indicate 20 μm. (C) The numbers of cell corpses. Data are presented as mean ± SD. *n*, number of animals scored; NA: not applicable due to embryonic-lethal phenotype. (D) DIC images of a wild-type 4-fold stage embryo (a) and *rab-7(ok511)* (m^−^z^−^) embryos that arrest prior to elongation (b), at 1.5-fold stage (c), and at 4-fold stage (d). *rab-7(ok511)* (m^−^z^−^) embryos were synchronized and were scored 12 h after being laid. The percentage of each class of phenotype is labeled. Arrows indicate cell corpses. Arrowheads indicate granules. Scale bar indicates 10 μm. (E) DIC time-lapse recording of germ-cell death induced by γ-ray irradiation and their subsequent removal in the gonad of wild-type (a–e) and *rab-7(ok511)* (f–j) adult hermaphrodites. Scale bar indicate 5 μm. (k) Histogram indicating distribution of the durations of germ-cell corpses. *n*, number of cell corpses scored. Two animals were used for each genotype. (F) TEM images of a *rab-7(ok511)* (m^+^z^−^) gonad. (e), (c), and (g) Indicate a cross section (e) and its enlarged regions that contain germ-cell corpses (c) and (g). Traces of membranes corresponding to (e), (c) and (g) are shown in (a), (b) and (f), respectively. Further enlarged images of framed regions in (c) and (g) are shown in (d) and (h), respectively. White arrows indicate germ-cell membrane; black arrows indicate sheath cell membrane; white arrowheads indicate cell corpse membrane; white open arrowheads indicate the nuclear envelope of the cell corpse. Scale bars indicate 5 μm in (e); 1 μm in (c) and (g); and 250 nm in (d) and (h). DCC, dark cell corpse. (i) Percentages of engulfed cell corpses in the gonads of wild-type and *rab-7(ok511)* at 48 h post-L4. A superscript letter *a* (^a^) indicates data from [1].

Previously, whether C. elegans RAB-7 is involved in cell-corpse removal was unknown. To determine the function of RAB-7 in the degradation of cell corpses suggested by its specific phagosomal localization, we characterized *rab-7(ok511)*, a deletion allele obtained from the C. elegans Gene Knockout Consortium (http://www.wormbase.org). We confirmed the presence of a genomic deletion that eliminates the first three exons and a 265-bp upstream sequence of *rab-7* in this allele (Figure 3A). Since no additional in-frame ATG codon exists flanking or downstream of the site of deletion, *rab-7(ok511)* is likely a null allele.

Homozygous *rab-7(ok511)* (m^+^z^−^; where m represents the maternal gene product and z the zygotic gene product) embryos produced by *rab-7(ok511)/+* mothers develop normally and do not contain excess cell corpses (Figure 3C). However, after reaching the adult stage, their gonads contain a large number of germ-cell corpses (Figure 3B and 3C). In contrast, due to the swift removal of cell corpses, the gonads of wild-type hermaphrodites at the same age contain very few cell corpses despite the continuous occurrence of apoptosis events in the germline (Figure 3B and 3C).

The homozygous *rab-7* (m^−^z^−^) progeny of *rab-7(ok511)* (m^+^z^−^) animals display 98% embryonic lethality and arrest at various embryonic stages (Figure 3D), indicating an essential function for maternal RAB-7 during embryonic development. We further observed various developmental defects in *rab-7* (m^−^z^−^) embryos, including defects in the consumption of yolk and failure in hypodermal cell closure (Figures 3D(c) and S2, Text S1, and Video S2). During embryogenesis, 113 cells undergo apoptosis [12]. However, at late 4-fold stage, the final stage before hatching, no cell corpses can be found in wild-type embryos due to their prompt removal (Figure 3C and 3D(a)). By contrast, *rab-7* (m^−^z^−^) embryos that arrest at 4-fold stage contain many persistent cell corpses (Figure 3C and 3D(d)), again suggesting defects in their removal.

To verify that the persistent cell-corpse–like objects are indeed apoptotic corpses, the *ced-3(n717)* mutation, which blocks most, if not all, programmed cell deaths [26,27] was introduced into the *rab-7(ok511)* background. In *rab-7(ok511); ced-3(n717)* double mutants, no cell-corpse–like objects were observed in the m^+^z^−^ adult hermaphrodite gonads or m^−^z^−^ embryos (Figure 3C), confirming that the cell-corpse–like objects accumulating in *rab-7* mutant animals are apoptotic cells.

### *rab-7* Mutant Animals Are Defective in the Removal of Cell Corpses, Not the Execution of Programmed Cell Death

Accumulation of cell corpses may be caused by excessive apoptosis or defects in the removal of cell corpses. To distinguish between these two possibilities, we monitored the generation and duration of germ-cell corpses induced by γ-ray irradiation in *rab-7* (m^+^z^−^) adults (Materials and Methods). We chose a 2-h recording period starting at 3 h after irradiation, since in the wild-type germline, the number of cell corpses reaches its peak 4 h after irradiation [28]. Within this period, we observed similar numbers of germ cells undergoing apoptosis in wild-type and *rab-7(ok511)* animals (average ten and nine apoptotic cells, respectively). This result suggests that *rab-7(ok511)* mutants are no more susceptible to apoptosis stimuli than wild-type animals. On the other hand, we observed that in *rab-7* mutants, all germ-cell corpses analyzed persisted for a significantly longer period of time than those in wild-type animals (Figure 3E), indicating that *rab-7* mutant gonads are defective in the prompt removal of germ-cell corpses.

### Loss of *rab-7* Function Results in Specific Defects in the Degradation of Engulfed Cell Corpses

To further distinguish whether the persistent germ-cell corpses observed in *rab-7* mutants are results of defects in engulfment or degradation, we analyzed germ-cell corpses and their surroundings using TEM (Materials and Methods). Apoptotic germ cells cellularize from the germline syncytium and are quickly engulfed by gonadal sheath cells, a single layer of somatic cells that wrap around germline syncytium [27]. We analyzed serial sections that span the entire diameter of each cell corpse to determine whether a cell corpse is totally inside a sheath cell. In *rab-7(ok511)* mutants, all germ-cell corpses observed in three different gonad arms were engulfed by gonadal sheath cells (Figure 3F). The accumulation of engulfed germ-cell corpses suggests that the degradation, and not the engulfment, of cell corpses is blocked. In addition, in *rab-7* mutants, 90% of excessive germ-cell corpses are labeled with 2xFYVE::GFP from engulfing cells, respectively (Figure 2B and 2C), again indicating that they persist inside phagosomes.

Under TEM, in 59% of corpses in *rab-7* mutants, the nuclear envelope and plasma membrane were readily distinguishable, and the nucleolus appeared as a distinct patch with high electron density (Figure 3F(c) and 3F(e)). These well-preserved cellular characteristics suggest a defect in the initiation of cell-corpse degradation. In the rest of cell corpses, the electron density of osmium tetroxide–stained sections was greatly enhanced, and it was impossible to distinguish nucleoli or any intracellular membranous structures (Figure 3F(e) and 3F(g)), suggesting that a certain degree of phagosome maturation had occurred.

In 1.5- and 2-fold stage *rab-7* (m^−^z^−^) embryos, 78% of cell corpses are labeled with the PI(3)P marker (Figure 2A and 2C), indicating that they are inside phagosomes. Thus, somatic cell corpses in *rab-7* embryos, like germ-cell corpses, appear to be engulfed efficiently yet remain undegraded inside engulfing cells. We refer to the accumulation of engulfed cell corpses as a cell-corpse degradation defective (Ded) phenotype.

### The *rab-7* (m^+^z^−^) Adults Are Relatively Normal in Endocytosis and Lysosomal Functions

**Figure 4:**
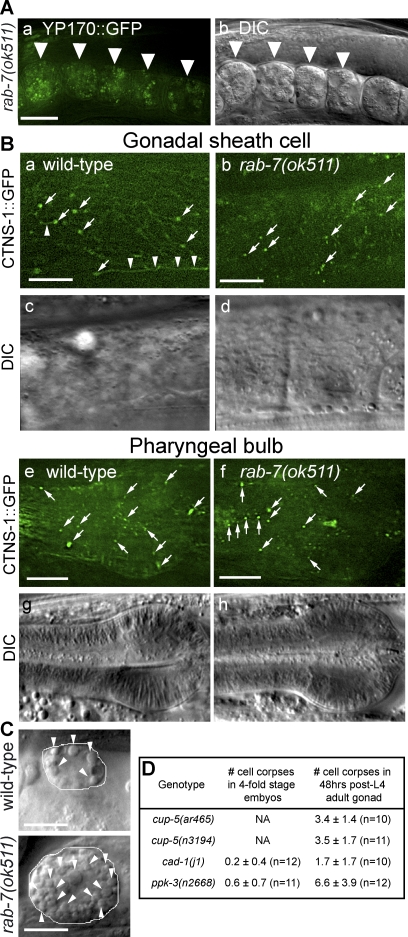
Yolk Uptake and Lysosome Morphology in *rab-7(ok511)* (m^+^z^−^) Adults Are Relatively Normal Anterior is to the left, and ventral to the bottom. (A) Epifluorescence and DIC images of the uterus of a *rab-7(ok511)* mutant expressing YP170::GFP. Arrowheads indicate fertilized oocytes. Scale bar indicates 20 μm. (B) Epifluorescence and DIC images of gonads and pharyngeal bulbs in wild-type ([a] and [b], and [e–f], respectively) and *rab-7(ok511)* (m^+^z^−^) ([c] and [d], and [g] and [h], respectively) adult hermaphrodite that express P*_ced-1_ ctns-1::gfp.* Scare bars indicate 10 μm. Arrows indicate CTNS-1(+) puncta. Arrowheads indicate tubular structures. (C) DIC images of coelomocytes in wild-type and *rab-7(ok511)* (m^+^z^−^) adult hermaphrodite. Scale bar indicates 10 μm. Coelomocytes are highlighted by white lines. Arrowheads indicate coelomocyte granules. (D) The number of cell corpses detected by DIC in embryos and adult gonads. Data are presented as mean ± SD. *n*, number of animals scored; NA, not applicable due to abnormal embryonic morphology.

To examine whether the defects in the degradation of germ-cell corpses are an indirect consequence of defects in endocytosis, lysosome biogenesis and function, and/or other vesicle transport events that might be affected by the loss of *rab-7* function, we analyzed a number of relevant aspects in *rab-7(ok511)* (m^+^z^−^) adult hermaphrodites, which display a strong Ded phenotype, but develop normally and appear normal in general morphology (see above). To examine possible defects in exocytosis and endocytosis, we first characterized the delivery of yolk to oocytes. In adults, yolk, a lipoprotein complex, is secreted from intestinal cells and transported to the gonad where it is internalized by oocytes through receptor-mediated endocytosis [29]. By monitoring YP170 (a yolk component)::GFP [29], we observed that just as in wild type [1], 100% of fertilized embryos inside *rab-7(ok511)* (m^+^z^−^) adult hermaphrodite gonads contain internalized YP170::GFP (Figure 4A). This result indicates that in these adults, the exocytosis and the subsequent endocytosis of yolk into oocytes are both normal. The accumulation of germ-cell corpses thus is unlikely to be a secondary consequence of a general endocytosis and/or exocytosis defect.

To detect lysosomes in *rab-7* mutants, we developed a specific fluorescent lysosomal marker, CTNS-1, tagged with GFP or mRFP1 at its C-terminus. CTNS-1 is the C. elegans homolog of human lysosomal cystine transporter cystinosin [30,31]. CTNS-1 colocalized to cytoplasmic puncta with RAB-7 and LMP-1, two proteins reported to localize to lysosomes, indicating that CTNS-1 was primarily localized to lysosomal compartments (Figure S3 and Text S1).

We compared the number and morphology of CTNS-1::GFP(+) particles in several tissues in wild-type and *rab-7* (m^+^z^−^) adult hermaphrodites and did not observe any obvious difference between these strains (Figure 4B). Recently, it was reported that in *rab-7(ok511)* (m^+^z^−^) adult hermaphrodites, the acidification of lysosomal compartments in coelomocytes, special scavenger cells that are highly active in endocytosis, was normal [32]. These lines of evidence suggest that the biogenesis and functions of lysosomes in *rab-7(ok511)* (m^+^z^−^) adult animals are relatively normal, probably owing to the residual wild-type maternal gene activity. By contrast, in *rab-7(ok511)* (m^−^z^−^) embryos that lack both maternal and zygotic gene activity, we detected severe defects in lysosomal functions (Figure S2 and Text S1). We thus conclude that the accumulation of engulfed cell corpses observed in the gonads of *rab-7(ok511)* (m^+^z^−^) adult animals is unlikely a secondary consequence of general defects in lysosome biogenesis or function.

We observed that in *rab-7(ok511)* (m^+^z^−^) hermaphrodites, coelomocyte vacuoles, which represent endocytic compartments [33], appeared the same size as in wild type, although they accumulated in a larger number (Figure 4C). This is in sharp contrast to the phenotypes observed in typical lysosome biogenesis mutants, such as *cup-5* or *ppk-3* mutants, on which coelomocyte vacuoles are enlarged [25,33]. On the other hand, mutations in *cup-5* or *ppk-3* resulted in no or very weak Ded phenotypes (Figure 4D). In addition, in *cad-1* mutants, in which the activity of aspartyl protease cathepsin D, a lysosomal hydrolase, was severely reduced [34], no Ded phenotype was observed (Figure 4D). These results imply that in addition to its function in regulating lysosome biogenesis, RAB-7 mediates an independent function related to phagosome maturation.

### The Active, GTP-Bound RAB-7 Functions in Engulfing Cells to Promote the Degradation of Cell Corpses

**Figure 5:**
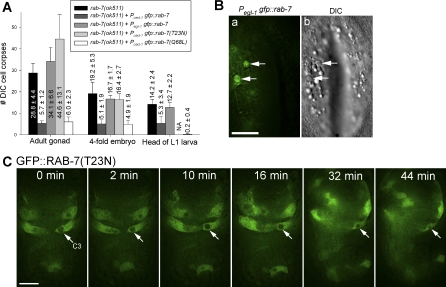
GTP-Bound RAB-7 Acts in Engulfing Cells to Promote Cell-Corpse Degradation (A) The numbers of DIC(+) cell corpse in *rab-7(ok511)* (m^−^z^−^) animals at different stages that express different transgenes. Data are presented as mean ± SD and are labeled above the bars. NA, not applicable due to 100% embryonic lethality. At least ten animals were scored for each datum. (B) Epifluorescence (a) and DIC (b) images of a 4-fold stage *rab-7(ok511)* (m^−^z^−^) embryo that expresses *P_egl-1_ gfp::rab-7*. Arrows indicate dying cells that express GFP::RAB-7. Scale bar indicates 10 μm. (C) RAB-7(T23N) is not localized to phagosomal surfaces. Time-lapse images of a wild-type embryo expressing P*_ced-1_gfp::rab-7(T23N)*, starting from the completion of engulfment (0 min). Arrows indicate the phagosome containing C3. Scale bar indicates 10 μm.

GFP::RAB-7, when produced in engulfing cells (P*_ced-1_gfp::rab-7*), efficiently rescued the Ded phenotype in *rab-7(ok511)* mutant embryos, L1 larvae, and adult gonad (Figure 5A). On the other hand, a P*_egl-1_gfp::rab-7* construct, which specifically expressed GFP::RAB-7 in apoptotic cells (Figure 5B) [35], did not display any rescuing activity (Figure 5A). These observations suggest that *rab-7* is likely to act in engulfing cells, not dying cells, to promote cell-corpse degradation.

Rab proteins are known to cycle between the GTP-bound, active state and the GDP-bound, inactive state [36]. A T22N mutation of mammalian Rab7 (Figure S1A) results in a great decrease of its in vitro GTP-binding activity [37]; on the other hand, the Q67L mutation (Figure S1A) decreases its GTP hydrolysis activity, and thus locks it in a GTP-bound and active state [38,39]. We analyzed these two mutations for their effects on RAB-7 function. The transgene P*_ced-1_gfp::rab-7(T23N)* did not rescue the Ded phenotype in *rab-7(ok511)* mutants (Figure 5A), indicating that GTP binding is essential for RAB-7 function. On the other hand, P*_ced-1_gfp::rab-7(Q68L)* displayed a rescuing activity similar to (in gonads and embryos) or stronger than (in L1 heads) that of P*_ced-1_gfp::rab-7* (Figure 5A), suggesting that like many other small GTPases, GTP RAB-7 is an active form in cell-corpse degradation.

In engulfing cells, GFP::RAB-7(Q68L), like GFP::RAB-7, was enriched on the surface of phagosomes (unpublished data). In contrast, GFP::RAB-7(T23N) failed to accumulate to phagosomes (Figure 5C). These results indicate that only the GTP-bound, active form of RAB-7 is recruited to phagosomal surfaces and establish a correlation between the localization and function of RAB-7 on phagosomal surfaces.

### The Loss of *rab-7* Function Does Not Affect the Presentation of PI(3)P on Phagosomal Surfaces or the Incorporation of Early Endosomes to Phagosomes

We observed that in both embryos and gonads, the percentage of cell corpses labeled by 2xFYVE::GFP circles were similar between wild type and *rab-7* mutant (Figure 2), indicating that the enrichment of PI(3)P on phagosomal surfaces is normal in *rab-7* mutant. In addition, in embryos that in engulfing cells expressed HGRS-1::GFP, a marker for early endosomes [1], both the percentage of phagosomes labeled with HGRS-1::GFP (39% and 42%, respectively) and the intensity of GFP on phagosomal surfaces were similar between wild type and *rab-7(ok511)* (Figure 2A and 2C) [1]. This result indicates that the function of RAB-7 is not essential for the delivery of early endosomes to phagosomes.

### The Incorporation of Lysosomes to Phagosomes Is Severely Defective in *rab-7* Mutants

**Figure 6:**
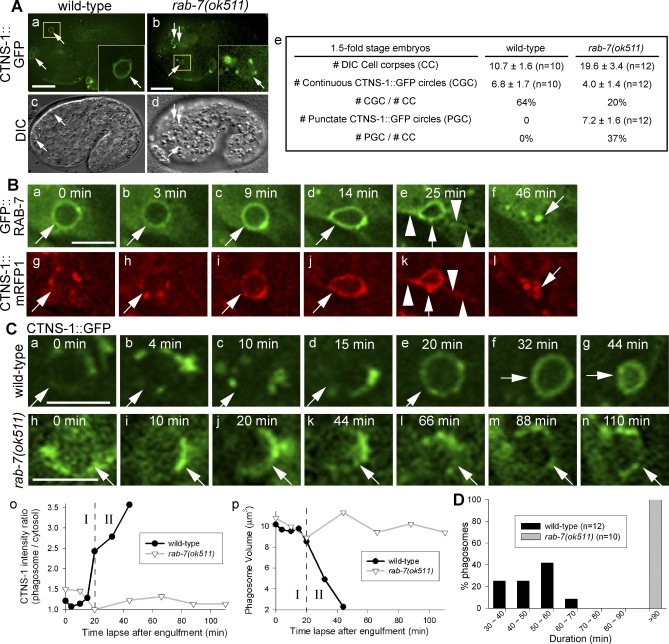
*rab-7* Is Required for Both the Recruitment and Fusion of Lysosomes to Phagosomes (A) CTNS-1::GFP around cell corpses in 1.5-fold wild-type and *rab-7(ok511)* (m^−^z^−^) embryos. Insets represent 3-fold enlargement of the boxed regions. (e) The number of DIC(+) cell corpses and CTNS-1::GFP circles around cell corpses in 1.5-fold embryos are presented as mean ± SD. *n*, number of animals scored. Scale bars represent 10 μm. (B) Time-lapse images of epifluorescence signals on the C2 phagosome (arrows) in a wild-type embryo coexpressing P*_ced-1_ ctns-1::mrfp1* and P*_ced-1_ gfp::rab-7.* Scale bar indicates 5 μm. “0 min” represents when GFP::RAB-7 is first recruited to C2. Arrowheads indicate tubular structures extended from phagosomes. (C) Time-lapse images of CTNS-1::GFP signal on the C1 phagosome (arrows) in wild-type and *rab-7(ok511)* (m^−^z^−^) embryos. “0 min” represents when the nascent C1 phagosome is formed. Scale bars indicate 5 μm. (o) and (p) Quantitative data measured from images (a–n), including CTNS-1::GFP intensity ratio (phagosome/cytosol) and phagosome volume, are plotted over time. The two phases of CTNS-1 localization pattern are labeled as I and II. (D) Histogram indicating the distribution of the durations of phagosomes in wild-type and *rab-7(ok511)* (m^−^z^−^) embryos measured using CTNS-1::GFP as a reporter. *n*, number of phagosomes scored.

In wild-type embryos, 64% of cell corpses are labeled with CTNS-1::GFP on their surfaces (Figure 6A), indicating that lysosomes are incorporated into phagosomes. We monitored the delivery of CTNS-1::GFP signal to phagosomal surfaces in both embryos that expressed P*_ced-1_ ctns-1::gfp* and embryos that coexpressed P*_ced-1_ gfp::rab-7* and P*_ced-1_ ctns-1::mrfp1.* The dynamic CTNS-1::GFP recruitment to phagosomes occurred in two distinct phases. During phase I, which started after the formation of nascent phagosomes and lasted approximately 20 min, a weak and punctate CTNS-1::GFP signal was observed on the surface of phagosomes (Figure 6B(g–i), 6C(a–d), and 6C(o)). During the following phase II, CTNS-1::GFP rapidly accumulated on a phagosome, and gradually evolved from multiple punctate spots to a smooth, continuous circle (Figure 6B(i–l) and 6C(d–g)). Following the rapid enrichment of CTNS::GFP signal, the volume of a phagosome quickly decreased (Figure 6B, 6C(o), and 6C(p)), indicating degradation of phagosomal contents. A phagosome remained CTNS-1::GFP(+) until it was completely degraded (Figure 6B and 6C). The accumulation of the CTNS-1::GFP signal is indicative of the recruitment of lysosomes to phagosomal surfaces, whereas the subsequent transition from punctate to continuous circles may represent the fusion of lysosomes to phagosomal membranes. The recruitment of RAB-7 to phagosomes took place earlier than the phase II CTNS-1 enrichment (Figures 1E and 6B), with an average interval of 13.4 ± 5.8 min (*n* = 9).

In *rab-7(ok511)* embryos, as in wild type, CTNS-1::GFP is localized to cytoplasmic puncta (Figures 6A). However, we observed severe defects in the incorporation of lysosomes into phagosomes. Most (65%) of all CTNS-1::GFP circles observed around cell corpses in 1.5-fold *rab-7(ok511)* embryos remain punctate (Figure 6A(e)). Time-lapse recording results indicated that the localization of CTNS-1::GFP to phagosomes was mostly arrested at phase I: the signal intensity on phagosomes remained low (Figure 6C(o)), suggesting a defect in the recruitment of lysosomal particles; furthermore, the signal observed on phagosomal surfaces remained in a punctate pattern for an extended period of time (>100 min) (Figure 6C(h–n)), suggesting a likely defect in lysosomes/phagosome fusion.

To quantify the degradation defect caused by the *rab-7(ok511)* mutation, we monitored the duration of CTNS-1::GFP-labeled phagosomes. The volume of phagosome C1 in wild-type embryo decreased 5-fold within 40 min of its presence, yet remained unchanged over 2 h in a *rab-7(ok511)* embryo (Figure 6C(p)), indicating a lack of digestion of phagosomal contents. In wild-type embryos, phagosomes disappeared within a period of 30–70 min after their formation; in *rab-7(ok511)* embryos, ten phagosomes monitored all lasted much longer than 90 min (Figure 6D). These results indicate that the defects in the incorporation of lysosomes to phagosomes caused by *rab-7* deletion are closely associated with the blockage of the degradation of phagosomal contents.

### RAB-7 Promotes the Extension of Tubular Structures for the Recruitment of Lysosomes to Phagosomal Surfaces, and the Fusion of Lysosomal Particles to Phagosomes

**Figure 7:**
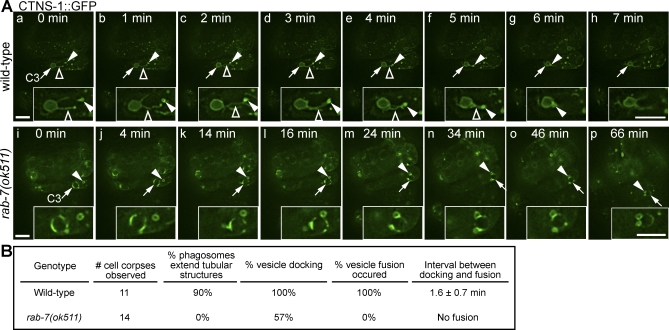
*rab-7* Mutants Are Defective in the Extension of Tubular Structures from Phagosomes and in the Recruitment and Fusion of Lysosomes to Phagosomes (A) As shown in Videos S3 and S4, time-lapse recordings of a CTNS-1::GFP–labeled lysosome particle (white arrowheads) being delivered to C3 phagosome (arrows) in wild-type (a–h) and *rab-7(ok511)* (m^−^z^−^) (i–p) embryos that express *P_ced-1_ ctns-1::gfp*. Open arrowheads indicate tubular structures. Scale bars represent 5 μm. (B) Quantitative data deduced from multiple time-lapse recording experiments similar to (A) that score the defects in the recruitment and fusion of lysosome to phagosomes in *rab-7(ok511)* (m^−^z^−^) embryos.

We further monitored individual CTNS-1::GFP(+) puncta over time and observed that in wild-type embryos, the CTNS-1(+) puncta were delivered to the surfaces of phagosomes through either direct encounters with phagosomes or attachment to membrane tubules originating from phagosomes. Tubules extending from phagosomal surfaces were frequently observed; these tubules were labeled with RAB-7 and CTNS-1 (Figures 6B and 7A), but not with PI(3)P (Figure 1D). As shown in Figure 7A and Video S3, within 6 min, a CTNS-1(+) punctum was seen to attach to such a tubule, and was carried to the phagosome by the retracting tubule. In *rab-7(ok511)* embryos, in contrast, we did not observe any tubular structures extending from phagosomes (Figure 7 and Video S4). The lack of extended tubules presumably leads to the decreased targeting of lysosomes to phagosomes, which subsequently results in insufficient delivery of lysosomal enzymes. Indeed, on the surface of 43% of phagosomes that we monitored in *rab-7(ok511)* embryos, no CTNS-1(+) particles were ever observed to be attached during the entire 100-min observation period (Figure 7B and Video S4). These observations suggest that RAB-7 is essential for the extension of membrane tubular structures from phagosomes, a process necessary for the efficient recruitment of lysosomes to the surface of phagosomes.

In wild-type embryos, after attaching to the phagosomal surface, lysosomal particles rapidly lost their distinct shape and became part of the phagosomal surface, a process likely suggesting the fusion of lysosomal particles to phagosomal membranes (Figures 6C(a–g) and 7A). In *rab-7(ok511)* embryos, however, in some cases, even after the attachment to phagosomal surfaces, the particles maintained their distinct shape for various amounts of time, sometimes greater than 50 min (Figure 7A(i–p) and Video S4), indicating an apparent lack of fusion. The defects in the attachment and possible fusion of individual puncta to phagosomes apparently correlate with the persistent punctate state and the low signal intensity of CTNS-1::GFP on phagosomal surfaces (Figure 6C(h–n)).

### Mutations in *ced-1*, *dyn-1*, and *ced-5* Delay the Degradation of Engulfed Cell Corpses

**Figure 8:**
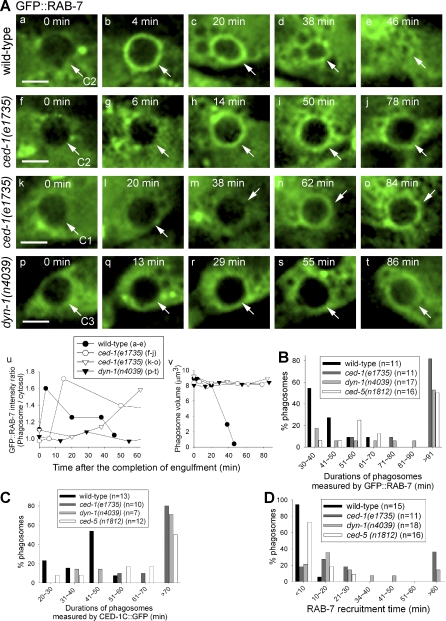
*ced-1* and *dyn-1* Mutants Are Defective in Recruiting RAB-7 to Phagosome Surface and in the Degradation of Cell Corpses (A) Time-lapse images (a–t) of GFP signal on phagosomal surfaces in developing embryos that express P*_ced-1_ gfp::rab-7*. Scale bars indicate 2 μm. Arrows indicate phagosomes. “0 min” represents when engulfment is just complete and phagosomes are detectable as dark holes inside the engulfing cell. Refer to Videos S1, and S5–S7 for the entire time-lapse series of whole-embryo images. (a–e) In a wild-type embryo, the phagosome quickly recruits GFP::RAB-7 and is degraded in normal time course. (f–o) In a *ced-1(e1735)* embryos, the degradation of both C2 (f–j) and C1 (k–o) phagosomes are severely delayed. C2 and C1 phagosomes are delayed to a different extent in the recruitment of GFP::RAB-7. (p–t) In a *dyn-1(n4039)* embryo, the C3 phagosome is blocked in both the recruitment of GFP::RAB-7 and degradation of the cell corpse. (u) The GFP::RAB-7 signal intensity ratios (phagosome/cytosol) measured from (a–t) are plotted over time. (v) Phagosome volumes measured from (a–t) are plotted over time. (B and C) Histogram distribution of phagosome durations in embryos using GFP::RAB-7 (B) and CED-1C::GFP (C) as reporters. Duration is measured from the formation of a nascent phagosome (indicated by a dark hole) until its degradation. *n*, number of phagosomes C1, C2, and C3 measured using time-lapse recording. (D) Histogram distribution of the time that it took to recruit RAB-7 to phagosomes, measured from the formation of a nascent phagosome (dark hole) until the GFP::RAB-7 signal intensity on the phagosomal surface is at least 1.2 fold as high as that in the host cell cytosol. *n*, number of phagosomes scored.

Previously, due to the lack of markers to label phagosomes and time-lapse imaging techniques to follow phagosome maturation, the *ced* genes acting in the two parallel pathways led by *ced-1* and *ced-5* were only implicated in the engulfment of cell corpses (Introduction). The diffused cytoplasmic localization pattern of GFP::RAB-7, in addition to its association to cytoplasmic puncta, allows us to follow the extension and closure of pseudopods during engulfment (Figure S4A and S4B). In addition, a phagosome can be distinguished by the enriched GFP::RAB-7 signal on its surface (Figure 8A(b)), or even before being labeled by GFP::RAB-7, recognized as a dark, GFP(−) hole inside a GFP::RAB-7(+) engulfing cell (Figure 8A(a)). To further study whether the engulfment and degradation of cell corpses employ common mechanisms, we examined the kinetics of the engulfment and degradation in *ced-1*, *dyn-1*, and *ced-5* mutant embryos using time-lapse imaging. *ced-1* and *ced-5* mutant embryos are viable [13]. Although *dyn-1(n4039)* embryos undergo developmental arrest, the arrest point is at 4-fold stage (>620 min after first cleavage) [1]. Thus our time-lapse recording, performed between 300–420 min after first cleavage, was not affected by the arrest.

We monitored the engulfment of cell corpses C3 in *ced-1(e1735)*, *dyn-1(n4039)*, and *ced-5(n1812)* null mutant embryos [1,15,40] that express P*_ced-1_ gfp::rab-7*. We demonstrated that these three mutants displayed distinct and severe defects in the initiation and/or the extension of pseudopods around cell corpses (Figure S4B–S4E and Text S1), consistent with the well-characterized functions of these proteins in initiation of engulfment. Interestingly, in only 19%–25% of single-mutant embryos, C3 remained unengulfed for longer than 100 min; in the other embryos, C3 were eventually engulfed (Figure S4E). In addition, in 1.5-fold stage *ced-1* and *ced-5* mutant embryos, approximately 20%–30% of DIC(+) cell corpses appeared as either dark holes or GFP-labeled phagosomes inside engulfing cells (Figure S4F and S4G), indicating that these cell corpses were engulfed, but not yet degraded.

The partial defects in engulfment allowed us to monitor the degradation of those cell corpses that were engulfed in *ced-1*, *dyn-1*, and *ced-5* embryos. In wild-type embryos, the duration of a phagosome, which was the total time period that it existed as a dark hole (nascent phagosome) and then as a GFP::RAB-7(+) circle, was 30–70 min (Figure 8A(a–e) and 8B). In *ced-1*, *dyn-1*, and *ced-5* mutants, however, the duration varied within a wider range, with a distinct population of phagosomes that lasted longer than 90–100 min, the time limit of our time-lapse recording due to the vigorous body movement of embryos occurring after reaching approximately 440 min after first cleavage (Figure 8B). The volume of the phagosomes belonging to this population did not reduce throughout the observation period (Figure 8A(v)), indicating a severe defect in the degradation of phagosomal content. This defect is further confirmed using CED-1C (the intracellular domain of CED-1)::GFP, another reporter that is evenly distributed in the cytoplasm of engulfing cells and allows the recognition of phagosomes as GFP(−) holes (Figures 8C and S5). The above observations confirm the defects of *dyn-1* mutants in phagosome maturation, which was observed before using different assays [1], and further reveal novel functions for *ced-1* and *ced-5* in the degradation of apoptotic cells, a step downstream of engulfment.

### *ced-1*, *dyn-1*, and *ced-5* Mutants Are Defective in the Recruitment of Lysosomes to Phagosomes

**Figure 9:**
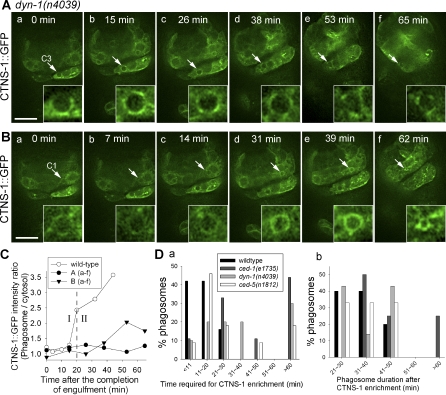
The Incorporation of CTNS-1–Labeled Lysosomes to Phagosomes Is Delayed to Different Extents in *ced-1*, *dyn-1*, and *ced-5* Mutants (A and B) Time-lapse images of one *dyn-1(n4039)* embryo expressing P*_ced-1_ ctns-1::gfp*. Anterior is to the top. Ventral faces reader. Scale bars indicate 10 μm. Arrows indicate phagosomes. “0 min” represents when engulfment is just complete. Insets represent 4-fold enlargement of the boxed regions. (A) The C3 phagosome fails to enrich for CTNS-1 within a period of 65 min and is not degraded. (B) The enrichment of CTNS-1 on the C1 phagosome is delayed to time point “31 min.” However, it proceeds to degradation after CTNS-1 enrichment at 31 min. (C) CTNS-1 intensity ratios (phagosomal surface/cytosol) measured from (A)(a–f) and (B)(a–f) are plotted over time. CTNS-1 intensity ratio in wild-type (Figure 6C(o)) is also included. The two phases of CTNS-1 localization on phagosome surface are labeled. (D)(a) Histogram distribution of the time period it takes for CTNS-1(+) particles to be enriched on C1, C2, and C3 phagosomal surfaces, which was measured from the formation of a nascent phagosome till the phagosome surface is labeled with continuous CTNS-1 signal. Number of phagosomes monitored: wild-type: 19; *ced-1*: 9; *dyn-1*: 10; *ced-5*: 14. (b) Histogram distribution of phagosome durations after CTNS-1 is enriched on phagosome surface. Number of phagosomes monitored: wild-type: 10; *ced-1*: 8; *dyn-1*: 7; *ced-5*: 15.

Since the formation of phagolysosomes is an essential step in the digestion of phagosomal contents (Figure 6), we first analyzed the incorporation of lysosomes to maturing phagosomes containing C1, C2, and C3 in *ced-1*, *dyn-1*, and *ced-5* embryos by monitoring CTNS-1::GFP. We observed that although the initial localization of weak CTNS-1::GFP(+) signal on phagosomal surfaces in phase I was relatively normal; the enrichment of CTNS-1::GFP signal expected for phase II was delayed or absent on the majority of phagosomes in all three mutants (Figure 9). In many cases, the weak CTNS-1::GFP signal on phagosomal surfaces was unstable (Figure 9A). The defects of lysosomes incorporation in *ced-1* and *dyn-1* mutant embryos are more severe than that in *ced-5* embryos (Figure 9D(a)), correlating with the levels of degradation defects displayed by the corresponding mutants (Figure 8B). Although the recruitment of CTNS-1(+) particles was severely delayed, once recruitment was complete, in most cases, the corresponding phagosome would disappear within 50 min (Figure 9B and 9D(b)). For instance, we observed different fates of two phagosomes in one *dyn-1(n4039)* embryo: the weak, initial CTNS-1 signal labeling the phagosome containing C3 failed to increase over time, and C3 remained undegraded after 65 min (Figure 9A), whereas the phagosome containing C1 was eventually degraded after a delayed enrichment of CTNS-1 signal (at 31 min) on its surfaces (Figure 9B). These results suggest that the inefficient incorporation of lysosomes into phagosomes is one of the major causes for the cell-corpse degradation defects observed in *ced-1*, *dyn-1*, and *ced-5* mutants.

### The Accumulation of RAB-7 on the Surface of Phagosomes Is Delayed or Blocked in *ced-1* and *dyn-1*, but Not *ced-5*, Mutants

Since RAB-7 activity is essential for the incorporation of lysosomes to phagosomes, we monitored the dynamic association of RAB-7 with phagosomes in *ced-1*, *dyn-1*, and *ced-5* mutants. In both wild-type and *ced-5* embryos, greater than 70% of phagosomes are labeled with a GFP::RAB-7 circle within 10 min after the completion of engulfment (Figure 8D). By contrast, in *ced-1* and *dyn-1* embryos, the recruitment of RAB-7 to phagosomal surfaces is delayed or blocked. The majority of phagosomes in *ced-1* (82%) and *dyn-1* (79%) mutants remained RAB-7(−) (detected as dark holes) for greater than 10 min (Figure 8A(f–t) and 8D, and Videos S5–S7). In particular, 35% and 17% of phagosomes in *ced-1* and *dyn-1* mutant embryos, respectively, remained RAB-7(−) for greater than 60 min (Figure 8A(k–t) and 8D); all 11 such phagosomes failed to reduce in size throughout the recording period (for instance, Figure 8A(p–t)). These phenotypes indicate that (1) the recruitment of RAB-7 to phagosomal surfaces is essential for phagosome maturation, and (2) the recruitment is inefficient in *ced-1* and *dyn-1* mutants but relatively normal in *ced-5* mutants.

We examined the localization of RAB-7(Q68L) in *ced-1(e1735)* mutant embryos and found that the recruitment of RAB-7(Q68L) to phagosome surface is also delayed. However, the defect is less severe: whereas greater than 30% of phagosomes took more than 60 min to recruit RAB-7 after engulfment completes, all phagosomes recruit RAB-7(Q68L) within 50 min, with the majority within 30 min (Figure S6). These results suggest that enhancing GTP binding appears to alleviate the defect of RAB-7 recruitment caused by the loss of *ced-1* function.

### Mutations in *ced-1*, *ced-6*, and *dyn-1* Delay the Presentation of PI(3)P on Phagosomal Surfaces

**Figure 10:**
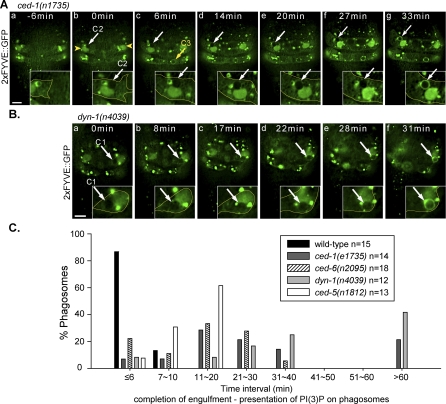
The Appearance of PI(3)P on Phagosomal Surfaces Is Significantly Delayed in *ced-1*, *dyn-1*, and *ced-6*, but Not *ced-5*, Mutants (A and B) Time-lapse recordings of a *ced-1(e1735)* (A) and a *dyn-1(n4039)* (B) embryo, expressing P*_ced-1_ 2xFYVE::gfp*. Anterior is to the top. Ventral faces readers. Arrows indicate phagosomes. Arrowheads indicate the nuclei of migrating hypodermal cells. Scale bars indicate 5 μm. “0 min” represents the first time point when engulfment is complete. The inset inside each panel is a 2-fold enlargement of the region around the white arrow, with the shape of the engulfing cell highlighted. In both (A) and (B), the appearance of PI(3)P on phagosome surface is delayed. (C) Histogram distribution of the PI(3)P presentation time in different backgrounds. The time is defined as the interval between the completion of engulfment and the first appearance of PI(3)P on phagosomal surfaces. Data are collected through time-lapse recording of the C1, C2, and C3 phagosomes. *n*, number of phagosomes scored.

To identify additional events important for phagosomal maturation regulated by the CED-1 pathway, we monitored the presentation of PI(3)P on phagosomes, which occurs prior to the recruitment of RAB-7 in wild-type (Figure 1D), *ced-1(e1735)*, *ced-5(n1812)*, *ced-6(n2095)* (a strong loss-of-function allele) [41], and *dyn-1(n4039)* embryos using the 2xFYVE::GFP or ::mRFP1 markers. In time-lapse experiments, nascent phagosomes not yet labeled with 2xFYVE markers were distinguished as dark holes inside engulfing cells (Figure 10A(b)). We observed the delays of PI(3)P presentation to different extents in different mutant embryos. In wild-type embryos, all phagosomes were labeled with 2xFYVE::GFP within 10 min after the completion of engulfment, among which 87% were labeled within 6 min (Figures 1C and 10C). The *ced-5(n1812)* mutation resulted in a slight delay of the appearance of PI(3)P: on 62% of phagosomes, PI(3)P was detected within 11–20 min after engulfment (Figures 10C and S7C, and Video S8). *ced-6(n2095)* embryos displayed an intermediate delay of PI(3)P presentation (Figures 10C and S7A). The *ced-1* and *dyn-1* embryos displayed the most severe defects—only 14% and 8% of phagosomes, respectively, were labeled with PI(3)P within 10 min of engulfment (Figure 10A–10C, and Videos S9 and S10). In addition, PI(3)P was absent from 21% and 42% of phagosomes, respectively, during a period that lasted greater than 60 min, suggesting that the presentation of PI(3)P on the surface of these phagosomes was blocked (Figures 10C and S7B, and Video S10). These observations suggest that the severe delay and/or blockage of PI(3)P presentation in *ced-1* and *dyn-1* mutants might affect multiple downstream maturation events**.**

## Discussion

### C. elegans as a Genetic System for Studying the Mechanisms Controlling the Degradation of Apoptotic Cells

Previously, due to the lack of cellular markers, whether a cell corpse was engulfed could not be easily distinguishable in C. elegans embryos. We have developed a series of live-cell imaging–based assays and have provided the first real-time observation of the entire cell-corpse removal process in the context of a developing embryo. Using these assays, we also established the temporal sequence of multiple phagosome maturation events. These in vivo assays have revealed that C. elegans RAB-7 plays essential roles in the formation of phagolysosomes. Our study indicates that the degradation of apoptotic cells in C. elegans shares certain mechanistic features with that of opsonized foreign objects in mammalian macrophages.

**Figure 11:**
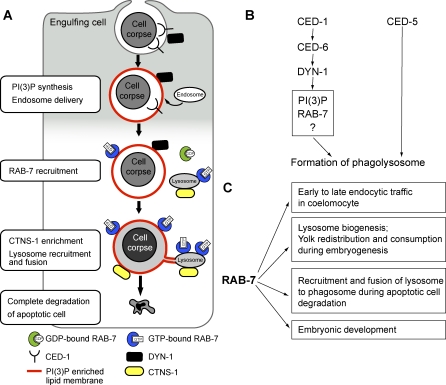
Signaling Pathways That Control the Degradation of Apoptotic Cells (A) Diagram summarizing the sequential molecular events that occur during the maturation of phagosomes containing apoptotic cells in C. elegans. (B) Diagram of the signaling pathways that promote the formation of phagolysosome. (C) A summary of the functions of C. elegans RAB-7.

Unlike TEM, which only detects the terminal phenotypes, the time-lapse recording system allows us to observe previously unknown defects in the dynamic process of phagosome maturation. We thus identified distinct roles of four C. elegans proteins, CED-1, CED-6, DYN-1, and CED-5, in the degradation of engulfed apoptotic cells (Figure 11). CED-1, CED-6, and CED-5 were only known for their functions in engulfment (see Introduction). Whether the mammalian homologs of these proteins are similarly involved in the maturation of phagosomes remains to be examined. Our findings suggest that C. elegans is a powerful model system for identifying additional genes and new mechanisms regulating phagosome maturation.

Mammalian macrophages elicit pro- or anti-inflammatory responses after engulfing pathogens, which are considered “foreign,” or apoptotic cells, which are considered “self,” respectively (see Introduction). The mechanisms behind these distinct responses remain largely unknown, and phagosomal surfaces are possible sites where these immune responses could be initiated. By studying the removal of apoptotic cells in C. elegans, we may find clues to those unknown mechanisms. For example, we observed that PI(3)P was enriched on C. elegans nascent phagosomes and persisted there until the complete digestion of phagosomal content. On the contrary, PI(3)P was reported to be transiently present on the surface of mammalian phagosomes containing opsonized objects, disappearing well before the completion of phagosome maturation [20]. These observations may reflect differences in the maturation of phagosomes with different contents.

### The Specific and Direct Functions of C. elegans RAB-7 for the Degradation of Apoptotic Cells

*C. elegans rab-7* was implicated in controlling cell survival based on the observation that *rab-7* (RNA interference [RNAi]) resulted in excessive germ-cell corpses in the adult hermaphrodite gonads [42]. Here, we report multiple lines of evidence demonstrating that the excessive germ-cell corpses observed in *rab-7* null mutants are unlikely caused by excessive cell death; rather, they are the result of defects in the degradation of engulfed apoptotic cells (Figure 3).

Mammalian Rab7 is involved in endocytic pathway and lysosome biogenesis [37,43–45]. Consistently, C. elegans RAB-7 is essential for endosomal trafficking and yolk consumption (Figure 11C) [29,32]. Our research provides several lines of evidence to suggest that the Ded phenotype of *rab-7* mutants is not merely a secondary consequence of the defects in the aforementioned functions, rather, RAB-7 plays a direct and independent role in phagosome maturation by acting on the surface of phagosomes. In adult *rab-7* (m^+^z^−^) mutant hermaphrodites, whereas intracellular trafficking and lysosome biogenesis are relatively normal due to the activity of *rab-7* maternal product, the degradation of engulfed cell corpses is severely impaired. In addition, mutations in *ced-1* and *dyn-1* cause strong and correlated defects in the recruitment of RAB-7 to phagosomal surfaces and in the formation of phagolysosomes, further supporting an independent role of RAB-7 on phagosomal surfaces.

Studies of the mammalian phagosomes containing infectious bacteria indicate that the functions and regulation of host-cell Rab7 during phagosome maturation are complex and diverse, and depend on the ingested bacteria species [46–48]. We found that regarding the degradation of apoptotic cells, which are distinct from latex beads, which are not degradable, and from infectious bacteria, which are foreign objects, RAB-7 displays both conserved and unique features. Mammalian Rab7 and its effector RILP were implicated in the extension of tubular structures from phagosomal surfaces, which attract late endosomes and lysosomes to phagosomes containing latex beads, based on the effects of the overexpressed dominant-negative forms of Rab7 and RILP [8]. We observed a similar extension of tubular structures from phagosomes and the recruitment of CTNS-1(+) lysosomal particles along those tubules in C. elegans (Figure 7). Furthermore, by analyzing *rab-7* null mutant embryos, we discovered that one of the functions of endogenous RAB-7 is to promote the extension of these tubules and the recruitment of lysosomes to phagosomes. We have also identified another function of RAB-7 in promoting the fusion of lysosomes to phagosomal surfaces. This fusion activity is consistent with the reported activity of Ypt7, the yeast counterpart of Rab7, in promoting the physical contact of vacuoles during homotypic vacuole fusion (reviewed in [49]), and indicates that RAB-7 is capable of mediating the fusion of organelles of different origins. In addition, dominant-negative Rab7 inhibits acidification of mammalian phagosomes that contain latex beads [8]. In contrast, we found that the null mutation of *rab-7* did not significantly affect the acidification of phagosomes containing apoptotic cells (Figure S8 and Text S1). Phagosomes containing apoptotic cells thus provide a new experimental system for further studying the mechanisms behind the functions and regulation of Rab7.

### How Is RAB-7 Recruited to the Phagosomal Surface—A Novel Signaling Pathway That Initiates the Formation of Phagolysosomes

In engulfing cells, RAB-7 exists in the cytoplasm both in a diffused form and in association with intracellular organelles, in particular lysosomes. The initial phagosomal appearance of RAB-7 occurs prior to the phagosomal acquirement of lysosomes, suggesting that free, cytosolic RAB-7 molecules are likely the primary source for phagosome-associated RAB-7. The GTP-bound form of RAB-7 is the active form recruited to and stably associated with phagosome, perhaps through a particular protein or protein complex that that mediates this association.

We have identified the novel functions of at least two genes, *ced-1* and *dyn-1*, for the timely recruitment of RAB-7 to phagosomes. This finding, together with the finding that RAB-7 is essential for the recruitment and fusion of lysosomes into phagosomes, indicate that the CED-1 pathway controls phagolysosome formation through regulating RAB-7 recruitment and function. Previously, we have reported that the phagocytic receptor CED-1 acts upstream of DYN-1 in the signaling pathway for engulfment [1]. We have also established that the transient localization of DYN-1 on the surface of phagocytic cups and phagosomes are both dependent on CED-1 function [1]. Thus we propose that CED-1 acts upstream of DYN-1 to control RAB-7 recruitment (Figure 11B).

The molecular mechanism for the recruitment of RAB-7 remains to be investigated. The observation that the recruitment of a GTP-bound, constitutively active form of RAB-7 to phagosomal surface was less affected than that of RAB-7 by the *ced-1* null mutation (Figure S6) may indicate that the CED-1 pathway may induce the formation of GTP-RAB-7 around phagosomal surfaces and the subsequent RAB-7/phagosome association. Alternatively, the CED-1 pathway may act in a more indirect manner, by recruiting or activating early signaling molecules that will further recruit RAB-7 (Figure 11A). We observed that PI(3)P was dramatically enriched on phagosomal surfaces prior to RAB-7. Furthermore, in *dyn-1*, *ced-6*, and *ced-1* mutants, the enrichment of PI(3)P on phagosomal surfaces was delayed. These observations suggest that PI(3)P might be related to the recruitment of RAB-7.

### The CED-1 Pathway Controls Multiple Important Events for Phagosome Maturation

The enrichment of RAB-7 on phagosomes is not the only phagosome maturation event regulated by the CED-1 signaling pathway. For instance, the recruitment of early endosomes to phagosomes, which is independent of RAB-7 (Figure 2), is dependent on CED-1, CED-6, and DYN-1 [1]. In addition, we observed that the rate of enrichment of PI(3)P on phagosomal surfaces is under the control of *ced-1*, *ced-6*, and *dyn-1*. PI(3)P attracts FYVE or PX domain–containing effectors, some of which play diverse roles during phagosome maturation, to phagosomal surfaces (reviewed in [50]). CED-1::GFP was observed to transiently cluster on nascent phagosomal surfaces for merely 9 min, in a time period that barely overlaps with that of PI(3)P and RAB-7 (Figure 1E). However, DYN-1 remains on phagosomal surfaces for a longer period (18 min). CED-1 thus may establish an active state of phagosomal surfaces through the activation of DYN-1. The rapid accumulation of PI(3)P on the surfaces of nascent phagosomes is likely to reflect the local activation of a Class III PI(3) kinase [20], whose activity and/or localization on phagosomal surfaces may be controlled by the CED-1 pathway.

We thus propose that CED-1, CED-6, and DYN-1 act in a signaling pathway to control both the engulfment and the degradation of engulfed apoptotic cells, through regulating different downstream effectors (Figure 11B). On nascent phagosomes, or perhaps even before the phagocytic cup is enclosed, this pathway may actively prime the phagosomal membrane for the reception of multiple signaling molecules and sequential incorporation of intracellular vesicles (Figure 11A).

Although extensive studies suggest that nascent phagosomes possess multiple signaling molecules on their surfaces, the identity of the ultimate initial signal that triggers phagosome maturation remains unclear. An activated phagocytic receptor may well be the source of this signal. When in complex with extracellular ligands, phagocytic receptors were known to undergo certain conformational changes that lead to the activation of their intracellular domains [51], which in turn may act on the cytoplasmic side of phagocytic cups and phagosomes to recruit cytosolic factors. Our report that CED-1 initiates phagosomal maturation through activating downstream effectors suggests that phagocytic receptors for apoptotic cells in different organisms, including mEGF10, the human CED-1 homolog [52], and perhaps even phagocytic receptors for foreign pathogens, may act to regulate the downstream phagosome maturation events. Furthermore, by activating different phagocytic receptors, phagocytic targets that are “self” or “foreign” might thus initiate differential phagosome maturation processes.

### CED-5 Controls Phagosome Maturation through a Distinct Pathway

*ced-5* null mutants are defective in the recruitment of lysosomes to phagosomes. However, unlike *ced-1* or *dyn-1* mutations, null mutation in *ced-5* does not seem to affect the recruitment of RAB-7 on phagosomal surfaces, nor does it cause any severe defect in the presentation of PI(3)P on phagosomal surfaces. These results imply that CED-5 acts in a distinct pathway to control phagolysosome formation (Figure 11B), just like in engulfment (Figure 1B). During engulfment, the pathway led by CED-5 was shown to regulate cytoskeletal reorganization (reviewed in [11]). Recently, it was reported that cytoskeletal reorganization also plays an active role in phagosome maturation in mammalian cells [10,53]. It would be interesting to know whether CED-5 and other members of its pathway contribute to phagosome maturation through remodeling the cytoskeleton.

## Materials and Methods

### Mutations and strains.

C. elegans strains were grown at 20 °C as previously described [54]. The N2 Bristol strain was used as the reference wild-type strain. Mutations and integrated transgenes used are described by [55], except where noted otherwise: LGI, *ced-1(e1735)*; LGII, *rab-7(ok511)*, *cad-1(j1)* [34], *mIn1*[*dpy-10(e128) mIs14*[*myo-2::gfp* + *pes-10::gfp*]] [56]; LGIII, *cup-5(ar465)* [57]; *cup-5(n3194)* [58], *ced-6(n2095)*; LGIV, *ced-5(n1812)*, *ced-3(n717)*; LGV, *unc-76(e911*); LGX, *dyn-1(n4039)* [1], *ppk-3(n2668)* [25]. *rab-7(ok511)* was generated and provided by the C. elegans Gene Knockout Consortium in strain VC308 (genotype: *rab-7(ok511)/mIn1*). Transgenic lines were generated by microinjection [59]. Plasmids were coinjected with p76-18B [60] into *unc-76(e911)* mutants, and non-Unc progeny were identified as transgenic animals.

### Plasmid construction.

*rab-7*, *ctns-1(a)*, and *lmp-1* cDNAs were PCR amplified from a mixed stage C. elegans cDNA library (Z. Zhou and H. R. Horvitz, unpublished data) and confirmed to bear anticipated sequences. The T23N and Q68L mutations were introduced into *rab-7* cDNA using a QuikChange Site-directed Mutagenesis Kit (Stratagene). To generate P*_ced-1_gfp::rab-7*, the *gfp* coding sequence lacking the stop codon, obtained from plasmid pPD95.75 (a gift from A. Fire), was fused in frame to the ATG start codon of the *rab-7* coding sequence. The fusion gene was cloned into a pPD95.75-based plasmid, flanked by P*_ced-1_*, the 5.2-kb *ced-1* promoter [15], and the *unc-54* 3′ UTR on the 5′ and 3′ ends. The *mrfp1* cDNA is a gift from R. Tsien [21]. The P*_ced-1_lmp-1::mrfp1*, P*_ced-1_ctns-1(a)::gfp*, or P*_ced-1_ctns-1(a)::mrfp1* constructs were similarly generated by cloning the *lmp-1::mrfp1*, *ctns-1(a)::gfp*, or *ctns-1(a)::mrfp1* genes into the above plasmid, flanked by P*_ced-1_* and *unc-54* 3′UTR on the 5′ and 3′ ends, respectively. The *gfp::rab-7* fusion gene was also inserted between P*_egl-1_* and *egl-1* 3′ UTR to generate P*_egl-1_gfp::rab-7*.

### Determining the gene structure of *rab-7* in the *rab-7(ok511)* allele.

*rab-7* genomic DNA was PCR amplified using VC308 lysate as a template. The fragment amplified from the *ok511* allele, which was 742 bp shorter than wild type, was sequenced, and the exact boundaries of the deletion, tgtgatttttc... aattcctccaa, as reported by C. elegans Gene Knockout Consortium, was confirmed.

### Transmission electron microscopy.

Adult hermaphrodites 48 h past the mid-L4 stage were used for TEM analysis of germ-cell corpses and the neighboring gonadal sheath cells as previously described [1]. Serial 50-nm thin sections that cover the entire length of each cell corpse were analyzed to determine whether a germ-cell corpse is entirely inside the sheath cell.

### Quantifying the number of cell corpses.

Both somatic cell corpses in embryos and germ-cell corpses in the adult hermaphrodite gonads were scored under the Nomarski DIC microscope by their highly refractile appearance as previously established [1]. Embryonic stages were determined according to [1]. In brief, 1.5- and 2-fold stages are embryos whose body lengths are 1.5 or two times the length of an egg, respectively. Late 4-fold stages embryos are embryos whose bodies make three turns and contain fully developed pharyngeal grinders. Wild-type embryos at 1.5-, 2-, and early and late 4-fold stages correspond to embryos at approximately 420 min, 445 min, 660 min, and approximately 780 min past the first cleavage, respectively [1].

### Irradiation and time-lapse recording of the duration of germ-cell corpses.

Time-lapse analysis was performed on a DeltaVision system with Olympus IX70 microscope (Applied Precision). Adult hermaphrodites 24 h post-L4 stage were irradiated with a ^137^Cs source (Gammacell 1000, 8.33Gy/min) at 180Gy. 2.5 h after irradiation, samples were transferred onto NGM plates containing 1 mM Aldicarb; after 20 min, samples were mounted on a slide immersed in 0.8 mM Aldicarb solution and observed under DIC optics at 20 °C. Twenty serial *Z*-sections, at 1.0 μm/section, of the gonadal region were recorded every 3 min. Animals were closely monitored for viability during recording. The total number and the period of duration of cell corpses that occur during the first 2 h were scored.

### Fluorescence microscopy and time-lapse recording.

An Olympus IX70-Applied Precision DeltaVision microscope equipped with a Photometris Coolsnap digital camera and Applied Precision Softworx software were used to capture and deconvolve fluorescence images. To score the number of phagosomes labeled with a certain fluorescence marker, serial *Z*-section images of an entire embryo were recorded at 0.5-μm intervals, in 40 sections, and images were deconvolved before scoring. To monitor the process of engulfment and degradation of cell corpses C1, C2, and C3, as well as the subcellular localization of multiple cellular markers, recording of the ventral surface of embryos started at 310–320 min after first cleavage and lasted for 2 h in 1- or 2-min intervals. For each time point, eight serial *Z*-sections at a 0.5-μm interval were recorded. Signs such as embryo elongation and movement were closely monitored to ensure the embryo being recorded developed normally.

### Quantitative image analyses.

Fluorescence signal intensity was measured and images were analyzed using the ImageJ software. To calculate the ratio of signal intensity on the surface of phagosomes compared to that in the engulfing cell cytosol, the phagosomal surface and cytoplasm were individually defined using the “Free Hand” tool, and the modal value of each selected area was used in the ratio calculation. The enrichment of fluorescence signal on the phagosomal surface was considered significant when the ratio was greater than 1.2. To measure the volume (V) of phagosomes, we assumed they were spheres. The radius (R) of each phagosome was measured using the “Measuring” tool provided by ImageJ, and V is calculated as 1.333 π *R* ^3^. Under the described image-capture conditions, the pixel distance is 0.133 μm/pixel. To measure the size of yolk droplets, the threshold tool was adjusted to mark all droplets labeled with YP170::GFP in an embryo. The size of every droplet larger than 0.13 μm^2^ was analyzed and collected to generate the distribution histogram.

## Supporting Information

Figure S1 Characterization of C. elegans RAB-7 (A) Sequence alignment between C. elegans and human Rab7. Solid triangles indicate the residues mutated in RAB-7(T23N) and RAB-7(Q68L). An open triangle indicates the junction between third and fourth exons, where the chromosome deletion starts in *rab-7(ok511)* allele. Boxed region contains the prenylation motif. G, guanine binding motif; PM, phosphate/Mg^2+^ binding motif; RabF, Rab-specific regions.
(B and C) Epifluorescence and DIC images of wild-type animals expressing transgenic reporters P*_ced-1_ gfp::rab-7.* Scale bars indicate 10 μm. (B) GFP::RAB-7 is localized to cytoplasmic puncta (arrowheads) in an early embryo (a) and (d) and gonadal sheath cell (b) and (e). (c) and (f) are enlarged images of the framed region shown in (b) and (e). Ventral is to the bottom.
(C) GFP::RAB-7 labels the surfaces of phagosomes in a comma-stage embryo (a) and (d) and germ-cell corpses in a 48-h post-L4 adult gonad (c) and (f). (b) and (e) are enlarged images of the framed region shown in (a) and (d).
(6.2 MB TIF)

Figure S2 *rab-7(ok511)* (m^−^z^−^) Mutant Embryos Display Phenotypes That Suggest Defects in Lysosomal Functions (A) Epifluorescence and DIC images of a wild-type and a *rab-7(ok511)* (m^−^z^−^) mutant embryo that expresses YP170::GFP. Arrows indicate yolk droplets. Scale bars indicate 10 μm and 2.5 μm in the image and inset, respectively.
(B) Histogram distribution of yolk droplet sizes measured from images in (A). A total of 93 and 97 yolk droplets were scored in wild-type and *rab-7(ok511)* embryos, respectively.
(C) Epifluorescent images of the anterior region in wild-type and *rab-7(ok511)* (m^−^z^−^) embryos that express P*_ced-1_ ctns-1::gfp*. Scale bar indicates 10 μm. Arrows indicate cytoplasmic CTNS-1(+) puncta. Arrowheads indicate CTNS-1(+) tubular structures.
(5.7 MB TIF)

Figure S3 CTNS-1 Is a Specific Marker for Lysosomes (A) Alignment of human and C. elegans cystinosin (two isoforms), which are 47% identical. The two lysosomes-targeting motifs (I and II) and six transmembrane domains (TM) are labeled.
(B) Diagrams of P*_ced-1_ ctns-1::gfp* and the CTNS-1::GFP protein on lysosomal membrane. The C-terminus of the seven-transmembrane lysosomal cystine transporter resides in the cytosol.
(C and D) Epifluorescence and DIC images of embryos that express different reporters under the control of *P_ced-1_.* Solid arrowheads indicate cytoplasmic puncta. Scale bars indicate 10 μm. (C) CTNS-1::GFP expressed in a wild-type embryo is localized to cytoplasmic puncta in intestinal precursor cells. (D) In hypodermal cells of wild-type comma-stage embryos, CTNS-1 colocalizes with LMP-1 (a–c) or RAB-7 (d–f). Open arrowheads in the insets indicate plasma membrane labeled by LMP-1::mRFP1, but not CTNS-1::GFP. Insets represent 2-fold–enlarged images of the boxed regions.
(3.9 MB TIF)

Figure S4 Engulfment Defects Displayed by *ced-1*, *dyn-1*, and *ced-5* Mutants Detected by Time-Lapse Recording (A) Diagram illustrating the process by which hypodermal cell ABplaapppp engulfs C3 while extending towards the ventral midline (the location between ABplaapppp and ABpraapppp) and then fuses with ABpraapppp at the midline.
(B) Time-lapse images of the engulfing process in embryos that express P*_ced-1_ gfp::rab-7.* “0 min” represents when ABplaapppp started to fuse with ABpraapppp at the ventral midline (arrowheads). Scale bars indicate 2 μm**.** Arrows indicate C3. In the wild-type embryo, pseudopods extension started and completed before ABplaapppp fused with ABpraapppp. In *ced-1(e1735)* mutant, pseudopods extension did not start until fusion occurred. In *ced-5(n1812)* mutant, pseudopods extension started before cell fusion occurred, but did not complete until fusion was complete due to a slower pseudopod extension process.
(C) Mean time point of the initiation of engulfment in relation to hypodermal cell fusion. “0 min” represents when cell fusion occurred at the ventral midline. Error bars represent the standard deviation (SD).
(D) Histogram distribution of the time period of the engulfment process in wild-type and different mutant backgrounds, with GFP::RAB-7 as a reporter for pseudopod extension and closure. Engulfment time is defined as the period of time starting from pseudopodia budding to fully enclosing C3.
(E) Percentage of C3 that remain unengulfed throughout the time-lapse recording period (>100 min).
(F) Epifluorescence and DIC images of 1.5-fold stage wild-type and *ced-1(e1735)* embryo that express P*_ced-1_ gfp::rab-7.* Anterior is to the top. Ventral is to the left. Scale bars indicate 10 μm. Arrows indicate phagosomes.
(G) The number of cell corpses that are DIC(+), RAB-7(+), or RAB(−) yet are engulfed (as dark holes) in GFP::RAB-7(+) engulfing cells. Alleles tested: *ced-1(e1735)* and *ced-5(n1812)*. At least 12 embryos were scored for each data point. Data are presented as mean ± SD.
(5.6 MB TIF)

Figure S5 An Example of the Delayed Degradation of a Cell Corpse in a *ced-1* Mutant Embryo Detected by Time-Lapse Recording of the CED-1C::GFP (A) Diagram of the cell-corpse degradation process.
(B and C) Embryos all carry the P*_ced-1_ ced-1C::gfp* reporter gene. Anterior is to the top. Ventral faces readers. Arrows indicate cell corpses in DIC images and phagosomes in fluorescence images. Scale bars indicate 10 μm. (B) DIC and epifluorescence images of a wild-type embryo to show that phagosomes containing cell corpses can be observed as dark holes inside engulfing cells that express CED-1C::GFP. Arrowheads indicate the three hypodermal cells that have engulfed cell corpses C1, C2, and C3. (C)(a–l) Time-lapse images of the degradation process of C3 (arrows) in a wild-type and C2 (arrows) in a *ced-1(e1735)* mutant embryo. (m) Phagosome volumes measured in (a–l) are plotted over time.
(5.5 MB TIF)

Figure S6 Recruitment of RAB-7(Q68L) to *ced-1* Mutant Phagosome Is More Efficient Than That of RAB-7 Histogram distribution of recruitment time of GFP::RAB-7(Q68L) in *ced-1(e1735)* embryos, compared to GFP::RAB-7 (data from Figure 8D).
(319 KB TIF)

Figure S7 Examples of the Delayed Appearance of PI(3)P on Phagosomal Surfaces Observed in *ced-6*, *dyn-1*, and *ced-5* Mutants Time-lapse image series of three embryos in different mutant backgrounds, either coexpressing P*_ced-1_ ced-1::gfp* and P*_ced-1_ 2xFYVE::mrfp1* (A) or expressing P*_ced-1_ 2xFYVE::gfp* (B-C), starting at approximately 330 min after first cleavage. Anterior is to the top. Ventral faces readers. Scale bars indicate 5 μm. “0 min” represents the time point that nascent phagosomes (white arrows) first form. The inset inside each panel is a 2-fold enlargement of the region around the white arrow, with the shape of the engulfing cell highlighted.
(A) PI(3)P is not presented on the surface of the C1 phagosome (white arrows) until 18 min after engulfment is completed in a *ced-6(n2095)* embryo.
(B) PI(3)P is not presented on the surface of the C2 phagosome (white arrows) throughout the recording period of 95 min in a *dyn-1(n4039)* embryo. A yellow arrow indicated a C3 phagosome on which the appearance of PI(3)P is relatively normal.
(C) PI(3)P appears on the surface of the C1 phagosome (white arrows) 10 min after the completion of engulfment in a *ced-5(n1812)* embryo. A yellow arrow indicates a C3 phagosome on which the timing of the PI(3)P appearance was normal.
(7.5 MB TIF)

Figure S8 Phagosome Acidification in *rab-7(ok511)* Mutant Is Normal (A) The numbers of germ-cell corpses detected by DIC and 2xFYVE::GFP at 28 h post-L4 stage in wild-type and *rab-7(ok511)* (m^+^z^−^) adult hermaphrodites that express P*_ced-1_ 2xfyve::gfp*, in the absence or presence of irradiation (IR) treatment (Materials and Methods). Data are presented as mean ± SD. *n*, number of animals scored.
(B) Fluorescence and DIC images of irradiated gonads of wild-type and *rab-7(ok511)* (m^+^z^−^) adult hermaphrodites that are stained with Lysosensor Yellow/Blue (Materials and Methods). Arrows indicate cell corpses. Arrowheads indicate normal germ cells. Scale bar indicates 10 μm.
(C) Percentage of cell corpses that display different staining intensities in the gonads of wild-type and *rab-7(ok511)* (m^+^z^−^) adult hermaphrodites. *n*, number of cell corpses examined. At least three different gonadal arms were scored for each genotype.
(2.2 MB TIF)

Text S1 Supplementary Text of Protocols and Results Supplementary text includes the following sections:
1. Detection of phagosome acidification.
2. *C. elegans* RAB-7 is likely an ortholog of human Rab7.
3. *rab-7* mutant embryos display multiple developmental defects, including severe defects in lysosomal morphology and function.
4. CTNS-1 is a specific marker for lysosomes.
5. The dynamic defects in the engulfment of cell corpses in *ced-1*, *dyn-1*, and *ced-5* mutant embryos observed using time-lapse recording.
6. Phagosome acidification is normal in *rab-7(ok511)* mutant.
(67 KB DOC)

Video S1 The Dynamic Process of the Enrichment of GFP::RAB-7 on Phagosomal Surface and the Degradation of a Cell Corpse Epifluorescence images of GFP::RAB-7 in a wild-type embryo expressing P*_ced-1_gfp::rab-7* are assembled in a time-lapse movie. The interval between two consecutive frames is 2 min. The white arrow indicates C2. At the “0 min” time point, C2 was partially internalized into the engulfing cell. At “2 min,” C2 is completely engulfed and a nascent phagosome first formed. Anterior is to the top. Ventral faces the reader.
(6.1 MB MOV)

Video S2 The Development Process of a *rab-7(ok511)* (m^−^z^−^) Embryo Showing Multiple Phenotypes DIC images were recorded starting at the first cleavage. The first image of this movie is at 400 min after first cleavage. The interval between two consecutive frames is 2 min. Anterior is to the left. Judging by morphology at 400 min after first cleavage, the embryonic development before that stage was delayed. The movie also shows that this embryo failed to continue elongation after reaching 2-fold of body length. In addition, hypodermal cells failed to fully enclose at the head region and as a result, the head tissue leaked out of the soma.
(8.5 MB MOV)

Video S3 In a Wild-Type Embryo, CTNS-1(+) Particle First Docks onto the Tubular Structure Extended from Phagosome and Subsequently Fuse into Phagosome through Retraction of the Tubule The movie was captured with 1 min between each frame. The whole process, including both the recruitment and fusion, is less than 8 min.
(2.4 MB MOV)

Video S4 In a *rab-7(ok511)* (m^−^z^−^) Embryo, the Recruitment and Fusion of CTNS-1–Labeled Lysosomal Particles to Phagosomal Surface Are Both Defective This movie records the journey of a single CTNS-1(+) particle (arrow) towards a phagosome in a *rab-7* embryo expressing P*_ced-1_ ctns-1::gfp*. Images captured in 2-min intervals are shown. It took the CTNS-1(+) particle 16 min to be recruited to phagosomal surface, which is significantly longer than what was observed in wild-type embryos, indicating a defect in recruitment. In addition, this CTNS-1(+) particle failed to fuse to phagosomal membrane after attachment, in a period of 52 min, indicating a fusion defect.
(9.7 MB MOV)

Video S5 The Enrichment of RAB-7 on Phagosomal Surfaces Is Moderately Delayed in a *ced-1(e1735)* Embryo This embryo expresses P*_ced-1_ gfp::rab-7*. Arrows indicate phagosome C2. “0 min” is the time point when cell corpse C2 is half engulfed. Time-lapse images are shown in 2-min intervals. RAB-7 enrichment is not detected on the surface of C2 until the 20 min point. The volume of phagosome C2 does not decrease throughout the recording period, indicating a degradation defect. Note that engulfment of C2 is also mildly delayed.
(5.3 MB MOV)

Video S6 The Enrichment of RAB-7 on Phagosomal Surfaces Is Severely Delayed in a *ced-1(e1735)* Embryo This embryo expresses P*_ced-1_ gfp::rab-7*. Arrows indicate phagosome C1. “0 min” is the time point when phagosome C1 first forms. Time-lapse images are shown in 2-min intervals. RAB-7 enrichment is not detected on the surface of C1 until the 56 min point. The phagosome size does not decrease throughout the recording period.
(4.9 MB MOV)

Video S7 The Enrichment of RAB-7 on Phagosomal Surfaces Is Not Detected in a *dyn-1(n4039)* Embryo This embryo expresses P*_ced-1_ gfp::rab-7*. Arrows indicate phagosome C3. “0 min” is the time point when C1 is partially engulfed. Time-lapse images are shown in 2-min intervals. RAB-7 enrichment is not detected on the surface of C1 throughout the 96-min recording period. The phagosome size does not decrease throughout the recording period, either. Note that engulfment of C1 is also mildly delayed.
(5.1 MB MOV)

Video S8 The Enrichment of PI(3)P on Phagosomal Surface Is Only Slightly Delayed in a *ced-5(n1812)* Embryo This embryo expresses P*_ced-1_2xFYVE::gfp*. “0 min” is the time point that engulfment is just complete, indicated by the formation of a dark hole, which is the nascent phagosome, inside the engulfing cell. Time-lapse images are shown in 2-min intervals. Arrows indicate phagosome C1, on which PI(3)P enrichment occurs at the 10 min point.
(1 MB MOV)

Video S9 The Enrichment of PI(3)P on Phagosomal Surface Is Severe Delayed in a *ced-1(e1735)* Embryo This embryo expresses P*_ced-1_2xFYVE::gfp*. “0 min” is the time point that engulfment is just complete, indicated by the formation of a dark hole inside the engulfing cell. Time-lapse images are shown in 2-min intervals. Arrows indicate phagosome C2, the PI(3)P enrichment on which does not occur until the 31 min point.
(3.2 MB MOV)

Video S10 The Enrichment of PI(3)P on Phagosomal Surface Is Blocked in a *dyn-1(n4039)* Embryo This embryo expresses P*_ced-1_2xFYVE::gfp*. “0 min” is the time point that engulfment is just complete (when the dark hole is first detectable). Time-lapse images are shown in 2- or 3-min intervals. Arrows indicate phagosome C2, on which PI(3)P enrichment is not detected throughout the recording period of 95 min. C2 fails to decrease in size throughout the recording period.
(4.3 MB MOV)

### Accession Numbers

The GenBank (http://www.ncbi.nlm.nih.gov/Genbank) accession numbers for RAB-7, CTNS-1(a), and CTNS-1(b) are CAA91357, CAA88102, and CAD56564, respectively.

## Abbreviations

Ced: cell death abnormal
Ded: cell-corpse degradation defective
DIC: differential interference contrast
GFP: green fluorescent protein
mRFP: monomeric red fluorescence protein
TEM: transmission electron microscopy
