# Supplementary material for: Phagocytic Receptor CED-1 Initiates a Signaling Pathway for Degrading Engulfed Apoptotic Cells
Source: PLoS Biol. 2008 Mar 18;6(3):e61. doi: 10.1371/journal.pbio.0060061 (PMC2267821; doi:10.1371/journal.pbio.0060061)
Supplement: Supplementary file 1 [file pbio.0060061.sd001.doc]

**Phagocytic Receptor CED-1 Initiates a Signaling Pathway for Degrading Engulfed Apoptotic Cells**

Xiaomeng Yu^1^, Nan Lu^1^, and Zheng Zhou^1,2,3^

# Text S1

# Materials and methods

**Detection of phagosome acidification**

Adult hermaphrodites at 24 hrs post-mid L4 stage were irradiated on plate with a ^137^Cs source (Gammacell 1000, Atomic Energy of Canada Ltd.) at 180 Gy. Four hours after irradiation, gonads were dissected out of animals, placed on a glass slide and incubated in M9 solution [1] containing 5 mM Lysosensor Yellow/Blue DND-160 (Invitrogen Molecular Probes). The specimen was examined using a Zeiss Axioplan 2 compound microscope equipped with motorized stage and AxioCam MRm digital camera under the excitation and emission wavelengths of 360 nm and 535 nm, respectively. The ImageJ software (http://rsb.info.nih.gov/ij) was used to measure the signal intensity. Since the signal intensity of living germ cell nuclei is 10% higher than that of the germline syncytium, we defined “no or faint staining” when the ratio of signal intensity of germ cell corpses compared to germline syncytium was <=1.1, “intermediate staining” when the ratio was above 1.1 and below 2.0, and “bright staining” when the ratio was >= 2.0.

# Results

***C. elegans* RAB-7 is likely an ortholog of human Rab7**

*C. elegans* RAB-7, which is encoded by *rab-7*, shares 71% identity with human Rab7 (Figure S1A). Both proteins contain five RabF motifs and the predicted C-terminal prenylation site, the characteristic of Rab GTPases, as well as GTP-binding motifs and two “switch” regions shared by all Ras-like small GTPases [2,3]. These structural similarities, combined with the absence of additional close Rab7 homologues in the *C. elegans* genome, indicate that *C. elegans* RAB-7 is a likely ortholog of human Rab7.

***rab-7* mutant embryos display multiple developmental defects, including severe defects in lysosomal morphology and function**

*rab-7(ok511)* mutants display a maternal-effect embryonic lethal phenotype that indicates an essential function of *rab-7* for embryonic development. *rab-7(ok511)*(m^+^z^-^) embryos are viable and develop to adulthood, whereas *rab-7(ok511)*(m^-^z^-^) embryos are embryonic lethal (Emb) and can be separated into three classes based on their morphology: those that arrest before embryonic elongation (65.0%) (Class I, Figure 3D(b)), or between the comma and 1.5-fold stages (22.3%) (Class II, Figure 3D(c)); and those that complete body elongation and formation of the pharyngeal grinder (12.7%) (Class III) (Figure 3D(d)). Only 16% of Class III embryos hatch. Those that hatch all arrest at L1 larval stage.

We observed that Class II and III *rab-7(ok511*)(m^-^z^-^) embryos contained a large number of refractile granules (Figures 3D and S2), which were labeled with YP170::GFP and represented yolk droplets in sizes larger than that observed in wild-type embryos (Figure S2 A-B). In addition, in 1.5- to 2-fold stage embryos, in which yolk particles should have redistributed from all embryonic cells to primarily intestinal cells in wild-type background [4,5], the YP170(+) yolk droplets remain all over the body (Figure S2A). In these embryos, the CTNS-1::GFP(+) lysosomal puncta (see below) appear bigger than in wild-type and are present in a smaller number (Figure S2C). These phenotypes are consistent with previous reports that suggested *rab-7*(RNAi) impaired lysosomal functions, resulting in defects in the digestion and subsequent secretion of lysosomal contents [6,7]. We further observed that in Class II embryos, hypodermal cells failed to fully enclose in the head region; as a consequence, internal cells leaked out during the subsequent body elongation (Figure 3D(c) and Movie S2). This phenotype is similar to that displayed by those mutants defective in epidermis enclosure [8].

These results suggest that in *rab-7(ok511*)(m^-^z^-^) embryos, the lack of both maternal and zygotic *rab-7* activity results in severe defects in the removal of cell corpses, lysosomal functions, and embryonic development. In comparison, in *rab-7(ok511*)(m^+^z^-^) animals, the wild-type *rab-7* product inherited from the heterozygous mothers apparently is sufficient to support embryonic and larval development as well as the relatively normal morphology and lysosomal functions. However, the wild-type maternal *rab-7* product is still not sufficient for the removal of germ cell corpses (Figure 3 B-C), indicating that there is an additional aspect of RAB-7 function that is independent of its functions in the control of lysosomal biogenesis and functions.

# CTNS-1 is a specific marker for lysosomes

To develop a specific fluorescent lysosomal marker, we examined the subcellular localization of CTNS-1, the *C. elegans* homolog of human lysosomal cystine transporter cystinosin [9,10]. Cystinosin is a seven-transmembrane domain lysosomal protein whose C-terminal YXXφ (φ, hydrophobic residue) and YFPQA motifs in the third cytoplasmic loop are both essential for lysosomal localization (Figure S3A) [11]. Among the two existing splicing isoforms, *ctns-1(a)* encodes both motifs required for the lysosomal localization of cystinosin (Figure S3A). Since the topology of CTNS-1(a) predicts a C-terminal cytoplasmic tail, we tagged GFP or mRFP1 to the C-terminus of CTNS-1(a) and engineered these constructs under the control of P*_ced-1_* (Figure S3B). In wild-type embryos, CTNS-1(a)::GFP is primarily localized to cytoplasmic puncta (Figure S3 C-D). Furthermore, CTNS-1 co-localizes with RAB-7 and LMP-1, two proteins that were previously reported to localize to lysosomes [7,12-14], on the same puncta (Figure S3D). These results indicate that the CTNS-1-positive puncta are likely lysosomal particles. CTNS-1 does not seem to display additional plasma membrane localization as LMP-1 does, or additional diffused cytoplasmic pattern as RAB-7 does, thus is a more specific lysosomal marker than LMP-1 or RAB-7 (Figure S3D).

**The dynamic defects in the engulfment of cell corpses in *ced-1*, *dyn-1*, and *ced-5* mutant embryos observed using time-lapse recording**

In engulfing cells, GFP::RAB-7 was detected in the cytoplasm, and a portion of it is enriched on cytoplasmic puncta (Figures S1B). The portion that is evenly distributed in the cytoplasm was observed to label extending pseudopods as thin processes during engulfment. Using GFP::RAB-7 as a marker for pseudopods, we monitored the engulfment of cell corpse C3. In wild-type embryos, the hypodermal cell ABplaapppp extended pseudopods around C3 during its migration towards the ventral midline, and internalized C3 5-10 min before it fused with ABpraapppp (Figure S4 A-C). By contrast, in *ced-1(e1735)* embryos, although ABplaapppp appeared to contact C3 at the normal time point, the initiation of pseudopod extension was greatly delayed, sometimes even to the point after the fusion of ABplaapppp with ABpraapppp occurred (Figure S4 B-C). This particular phenotype was not observed in *dyn-1(n4039)* or *ced-5(n1812)* embryos, and indicates a unique defect in the function for an engulfing cell to recognize a cell corpse, which is consistent with the previously established role for CED-1 as a phagocytic receptor [15,16]. In addition, in *ced-1* mutants, the engulfment process, from the initiation of pseudopod extension to the full enclosure of a phagosome, took significantly longer periods than in wild-type embryos (Figure S4D). This particular phenotype is shared by both *dyn-1(n4039)* and *ced-5(n1812)* mutants (Figure S4D). In 19-25% of embryos carrying one of the three above mutations, C3 remained unengulfed > 100 min (Figure S4E). The frequencies that engulfment of C3 was blocked are consistent with the previous report that 23-29% of all embryonic cell corpses remain un-removed throughout embryogenesis in the *ced-1*, *dyn-1*, and *ced-5* mutant backgrounds [5], and suggest that cell corpse C3 represents such a cell corpse whose engulfment is affected to the same extent as the average of all embryonic cell corpses. Together, the above results confirm that although the *ced-1*, *dyn-1*, and *ced-5* mutants are defective in the engulfment process, a large number of cell corpses are engulfed eventually in each single mutant background, albeit slowly and less efficiently.

# Phagosome acidification is normal in *rab-7(ok511)* mutant

The gradual acidification of the phagosomal lumen to pH<4.5 is an important maturation event that allows for the activation of digestive enzymes that degrade phagosome contents [17]. To determine whether phagosomes containing apoptotic cells undergo a similar acidification process, we examined the pH status of phagosomes in living animals using Lysosensor Blue/Yellow DND-160 (Invitrogen Molecular Probes), a dual emission-wavelength dye that displays predominantly yellow fluorescence in an environment of pH5.0 or lower (Materials and Methods in Text S1). We induced germ cell apoptosis by γ-ray irradiation, and analyzed the pH of phagosomes inside gonadal sheath cells 4 hrs after irradiation to ensure that the phagosomes were at approximately the same stage of their maturation process.

By quantifying the total number of germ cell corpses and the percentage of those labeled with 2xFYVE::GFP in wild-type adult hermaphrodites, we learned that irradiation results in a 7-fold increase in the number of germ cell corpses, the majority of which (78%) were engulfed (Figure S8A). After irradiation, living germ cells display faint yellow signal of Lysosensor DND-160, while 77% of the germ cell corpses display intermediate or bright DND-160 yellow signal in wild-type animals (Figure S8 B-C). We thus conclude that *C. elegans* phagosomes containing apoptotic cells do undergo acidification during phagosomal maturation.

We detected intermediate or bright DND-160 signal in 68% of germ cell corpses in irradiated *rab-7(ok511)* adult hermaphrodites (Figure S8 B-C), indicating that the engulfed germ cell corpses in *rab-7* animals undergo a normal acidification process. In addition, a higher percentage of germ cell corpses display bright yellow signal in *rab-7(ok511)* mutants than in wild-type animals (Figure S8C), which might be a result of the prolonged duration of phagosomes in *rab-7* mutant animals.

**References**

1	Wood WB, the community of *C. elegans* Researchers (1988) The Nematode *Caenorhabditis elegans*. Cold Spring Harbor, NY: Cold Spring Harbor Laboratory.

2	Pereira-Leal JB, Seabra MC (2000) The mammalian Rab family of small GTPases: definition of family and subfamily sequence motifs suggests a mechanism for functional specificity in the Ras superfamily. J Mol Biol 301: 1077-1087.

3	Pereira-Leal JB, Seabra MC (2001) Evolution of the Rab family of small GTP-binding proteins. J Mol Biol 313: 889-901.

4	Bossinger O, Schierenberg E (1996) The use of fluorescent marker dyes for studying intercellular communication in nematode embryos. Int J Dev Biol 40: 431-439.

5	Yu X, Odera S, Chuang CH, Lu N, Zhou Z (2006) *C. elegans* Dynamin mediates the signaling of phagocytic receptor CED-1 for the engulfment and degradation of apoptotic cells. Dev Cell 10: 743-757.

6	Grant B, Hirsh D (1999) Receptor-mediated endocytosis in the *Caenorhabditis elegans* oocyte. Mol Biol Cell 10: 4311-4326.

7	Nicot AS, Fares H, Payrastre B, Chisholm AD, Labouesse M et al. (2006) The phosphoinositide kinase PIKfyve/Fab1p regulates terminal lysosome maturation in *Caenorhabditis elegans*. Mol Biol Cell 17: 3062-3074.

8	Chisholm AD, Hardin J (2005) Epidermal morphology. Wormbook ed. The *C. elegans* Research Community. doi/10.1895/wormbook.1.35.1. www.wormbook.org.

9	Town M, Jean G, Cherqui S, Attard M, Forestier L et al. (1998) A novel gene encoding an integral membrane protein is mutated in nephropathic cystinosis. Nat Genet 18: 319-324.

10	Kalatzis V, Cherqui S, Antignac C, Gasnier B (2001) Cystinosin, the protein defective in cystinosis, is a H(+)-driven lysosomal cystine transporter. Embo J 20: 5940-5949.

11	Cherqui S, Kalatzis V, Trugnan G, Antignac C (2001) The targeting of cystinosin to the lysosomal membrane requires a tyrosine-based signal and a novel sorting motif. J Biol Chem 276: 13314-13321.

12	Treusch S, Knuth S, Slaugenhaupt SA, Goldin E, Grant BD et al. (2004) *Caenorhabditis elegans* functional orthologue of human protein h-mucolipin-1 is required for lysosome biogenesis. Proc Natl Acad Sci U S A 101: 4483-4488.

13	Kostich M, Fire A, Fambrough DM (2000) Identification and molecular-genetic characterization of a LAMP/CD68-like protein from *Caenorhabditis elegans*. J Cell Sci 113: 2595-2606.

14	Chen CC, Schweinsberg PJ, Vashist S, Mareiniss DP, Lambie EJ et al. (2006) RAB-10 is required for endocytic recycling in the *Caenorhabditis elegans* intestine. Mol Biol Cell 17: 1286-1297.

15	Zhou Z, Hartwieg E, Horvitz HR (2001b) CED-1 is a transmembrane receptor that mediates cell corpse engulfment in *C. elegans*. Cell 104: 43-56.

16	Venegas V, Zhou Z (2007) Two alternative mechanisms that regulate the presentation of apoptotic cell engulfment signal in *Caenorhabditis elegans*. Mol Biol Cell 18: 3180-3192.

17	Vieira OV, Botelho RJ, Grinstein S (2002) Phagosome maturation: aging gracefully. Biochem J 366: 689-704.
